# Regenerating the food system: A proposed vision and guiding principles for regenerative, inclusive food systems (RIFS)

**DOI:** 10.1007/s13280-025-02319-1

**Published:** 2025-12-29

**Authors:** Sinéad O’Keeffe, Tewodros Tefera Amede, Bebe Omedo Bockline, Robert Kajobe, Thies Reemer, Ben Haggard, Jochen Froebrich, Marianna Siegmund-Shultze

**Affiliations:** 1 Wageningen Plant Research, Wageningen University and Research, Postbus 16, 6700 AA Wageningen, The Netherlands; 2 Stichting Wageningen Research (SWR Ethiopia), Addis Ababa, Ethiopia; 3 Faculty of Agriculture, Egerton University, P.O. Box 536 Egerton, Kenya; 4 Directorate of Graduate Training, Research and Innovation, Muni University, P.O. Box 725 Arua, Uganda; 5 Wageningen Environmental Research, Wageningen University and Research, Postbus 47, 6700 AA Wageningen, The Netherlands; 6 Regenesis Institute for Regenerative Practice, Santa Fe, NM USA; 7 Wageningen Social and Economic Research, Wageningen University and Research, Postbus 88, 6700 AB Wageningen, Netherlands

**Keywords:** Ecosystem health, Food security, Food systems, Inclusive, Place-based, Regenerative, Resilient livelihoods

## Abstract

The aim of this perspective is to further the discussion on regenerating our food system, by describing our future food system (FS) vision to be a regenerative, inclusive food system (RIFS) and providing guiding principles that work within an interactive dynamic framework to achieve this vision. The five RIFS principles outlined in this paper are intended to support and guide place-based processes for regenerating our FS. These are: (1) sense of place and purpose; (2) socio-ecological design for innovation; (3) building connections; (4) fair, just and inclusive transitions; and (5) design for renewal (it is a living process). The use of the five principles are at the heart of RIFS, supporting transformative, experiential learning and guiding the actions and responsibility of all actors involved in FS transformation towards RIFS. We argue that when all these principles occur in a transformation process, this provides the fertile ground for achieving a RIFS’s vision.

**Graphical abstract:**

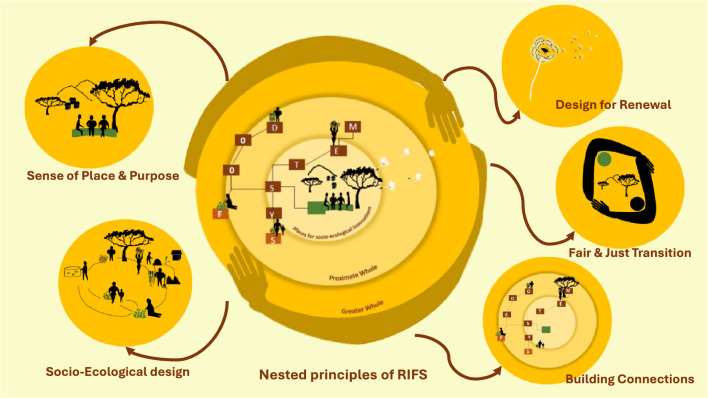

**Supplementary Information:**

The online version contains supplementary material available at 10.1007/s13280-025-02319-1.

## Introduction

“Food is what links humankind, now and in the future” (Fresco 2009). Food systems (FSs) are the interlinking network of people and places. It consists of actors whose activities coupled with biophysical resources provide us with our food and health, as well as dealing with our food waste (Berkum et al. 2018; de Steenhuijsen Piters et al. 2021; GAIN 2021). Culture and peoples’ values shape our FS (von Braun et al. 2021). They are fundamentally socio-ecological systems (SESs). The understanding of such SESs is that they are complex and adaptive, where people, communities and society are nested in nature, being influenced by and influencing each other, not separated into individual components (e.g. people, profit, planet) (Dahlberg 1993; Folke et al. 2016; Reyers et al. 2018).^1^ The FS, in this light, encompass much more than agriculture and food value chains, it includes all aspects of food and its conversion, consumption and decomposition. In other words, they comprise of different interacting sub-systems (e.g. agriculture, transport, processing, waste and water management) which are interlinked directly or indirectly with other sub-systems (e.g. energy, health, education, economics and governance). In short, FSs are webs of various interconnected social and ecological agents and processes (hence, SES). Therefore, FSs are multidimensional living systems influencing and being influenced by various nodes and interconnections in their networks (Berkum et al. 2018; Dahlberg 1993; Fresco 2009; Mang et al. 2016; Fresco et al. 2017; GAIN 2021).

The (ideal) outcomes of a FS is to ensure food and nutritional security for all people, while supporting decent livelihoods and well-being, as well as thriving ecosystems through which, resilience to shocks and stresses can be manifested (e.g. climate events, political events) (Ifejika Speranza et al. 2014; Du Preez et al. 2020). However, it is not only FS outcomes that are relevant, but also the way in which they are achieved. Developing resilient food systems is not only about the capacity to buffer and absorb shocks and stresses across the five capitals (i.e. human, social, natural, financial, and physical),^2^ it is also about our capacity and ability to have diverse connections to nature and to each other (self-organisation), as well as to learn from each other (capacity to learn) (Ifejika Speranza et al. 2014; Jacobi et al. 2018). Through supporting the capacities that characterise resilient SESs, the conditions required for food and nutritional security, decent livelihoods and thriving ecosystems can be achieved in a fair, just, and inclusive way (i.e. no one, including nature, is left behind) (Tribaldos and Kortetmäki 2022; Tschersich and Kok 2022). However, our current FSs are failing us, and these desirable outcomes are not being achieved (World Economic Forum 2017). Current FSs are not working for all, as increasing numbers of people are going hungry and are highly sensitive to system shocks, such as climate events, pandemics, economic recession and conflict. Fundamentally, many FSs lack long-term resilience and regenerative capacities and are rife with social injustices (Dahlberg 1993; Du Preez et al. 2020; de Steenhuijsen Piters et al. 2021; Tschersich and Kok 2022; De Bruin et al. 2024). In this perspective paper, we aim to advance the discussion on transforming our^3^ food systems by reimagining them to be regenerative and inclusive (RIFS) and developing five guiding principles to achieve this vision. These principles can be used to encourage and promote regenerative thinking and actions in the FS. The work conducted in this paper has been carried out as part of the REFOOTURE project (Adokorach et al. 2023a, b, c).

### Characterisation of extractive food systems

Over the last century, many food systems have morphed from diverse locally and regionally based networks to one of a global agro-industrial complex, which is an economically efficient system, but is highly extractive and heavily reliant on fossil fuels and chemical inputs (Dahlberg 1993, 1994; Sundkvist et al. 2005; Fresco 2009; Fresco et al. 2017; Loring 2021). As defined by Anderson and Rivera-Ferre (2021), *“extractive food systems view nature as something to be exploited by humans for profit and material gains, they do not view it as a living system with many intrinsic values upon which humans are dependent upon for our very existence”*. FS can have many forms of extractive components. These can range from heavily industrialised farming, as mentioned above, to subsistence agriculture and livelihoods, where people are heavily dependent on their own food production and in many cases have no choice but to carry out activities with deleterious effects on their supporting environment due to grinding poverty, social inequalities and poor governance (Amusan and Olutola 2017; Duguma et al. 2019; Ntuli et al. 2021). Therefore, there are a multitude of complex and interacting issues that are leading to the extractive behaviour found within a food system (Sanga and Koli 2023; Karami 2024). What is clear is that such activities and behaviours are at the centre of many of the grand societal challenges facing us today. This includes: climate change (e.g. contributing approx. a third of annual global GHG emissions) (IPCC 2019); water consumption (e.g. 70% of freshwater withdrawals are for agriculture, while 46% of the world population does not have access to safely managed sanitation) (Gruère et al. 2020; UN Water 2021); biodiversity loss (since 1970 the population sizes of mammals, amphibians, birds and reptiles have declined by approx. 68%) (Benton et al. 2021); toxicity and health (e.g. annually there are nearly 400 million cases of pesticide poisoning occurring, many in the global south) (Heinrich Böll Stiftung et al. 2022); malnutrition and hunger (e.g. globally 22% of children under 5 years of age were stunted and almost 3.1 billion people could not afford a healthy diet) (FAO et al. 2022); and land grabbing (e.g. land and land based wealth acquired due to the lack of, or different indigenous and cultural “land rights” being exploited) (IASG 2014; TNI 2013).

In many cases, extractive systems, such as the industrial one described above, operate usually on a global scale characterised by long supply chains, creating a disconnection between consumer and producer. These also consolidate power in the hands of a few dominant multinational corporations, comprising of many complex industry driven sub-systems (Fresco et al. 2017). In these systems, farmers are dependent on external inputs and have lost much of their market and resource autonomy (Anderson and Rivera-Ferre 2021; Van der Ploeg 2021; Tschersich and Kok 2022). In FSs which are more dependent on local and subsistence activities and less on external inputs, there is an awareness of the effect of extractive practices; however, the different actors are locked into this system of production, because these practices make food accessible and affordable to them (Amusan and Olutola 2017; Duguma et al. 2019; Ntuli et al. 2021). In general, FSs which are more extractive are also characterised by weakened links to ecosystems and with traditional ecological knowledge^4^ or indigenous knowledge being replaced by standardised knowledge (Reyes-García 2015). Thus, resulting in reducing the cognitive awareness of people in the food chain to recognise the signals of ecological feedback^5^ coming from unhealthy ecosystems producing our food (Larrick 1997; Sundkvist et al. 2005). This, in turn, reduces the regenerative capacities for learning and adaptation in order to build resilience and flexibility into the food system. While industrialising agriculture focuses predominantly on profit, as well as production and yields to feed the growing populations of humans, it does so at the cost of long-term sustainability. This is because it fails to contribute to the longer-term goals of building health, community resilience and vitality^6^ (Anderson and Rivera-Ferre 2021). There is, therefore, a radical and unprecedented need to disrupt, reconfigure, redesign, and regenerate our extractive food systems (Dahlberg 1993, 1994; Anderson and Rivera-Ferre 2021, Loring 2021). However, before this can be done, we must first reimagine what an alternative future food system could look like.

### Shifting the narrative from extractive to regenerative: reimagining our FS

Using the frame of “regenerative” FS has several advantages when compared to the term “sustainable” because it infers the need to regenerate both natural and social systems across generations (Dahlberg 1993). It urges us to use systems thinking to explore the multiple levels and dynamics of a FS across time (Caniglia and Mayer 2021). Furthermore, it redirects our focus to look at other aspects of the FS, not just on production, as it advocates for social inclusion, social justice, intergenerational and inter-species equity. The difference between regenerative FS and sustainable FS^7^ is the approach to engaging and interacting with ecosystems (Table 1). Sustainable FSs aim to limit or reduce the impact of food production and distribution, thus acknowledging there will be an extractive type of approach implemented. Regenerative on the other hand looks to work with nature to create a positive effect (e.g. using biomimicry or indigenous knowledge of the landscape systems). Through caring for nature, we can care for ourselves and for each other. In other words, in framing FS through a SES lens we can start to build greater livelihood resilience for communities with people and nature actively involved in the FS (Ifejika Speranza et al. 2014; Jacobi et al. 2018).

**Table 1:** Conceptual and comparative descriptions derived from the literature for degenerative, sustainable, and regenerative food systems (FS), to support a better understanding of potential differences. 1. Source of quotes is from Loring (2021), for the Maine lobster example, please see the discussion in Loring (2021) and 2. Pauly et al. (1998). 3. FAO (2018a). 4. Garí (2001), Coq-Huelva et al. (2017). Many of the descriptions have been derived from the papers and reports of: Dahlberg (1993), TNI (2013), Mang et al. (2016), Bellon-Maurel and Huyghe (2017), World Economic Forum (2017), FAO (2018a), Nyéléni (2019), Salembier et al. (2020), Anderson and Rivera-Ferre (2021), Benton et al. (2021), GAIN (2021), Loring (2021), Van der Ploeg (2021), FAO (2022), Heinrich Böll Stiftung et al. (2022)

Degenerative FS	Sustainable FS	Regenerative FS
**Mindset:** Reductionist tendencies, with the focus on the appropriation and extraction of resources (e.g. people, nature) driven by “strong economic incentives or subsidies, policies, or cultural norms… There is the assumption that the resources in question cannot be overharvested, or that they are so easily substituted that overharvest is irrelevant”^*1*^. Economic accumulation by the few and continuous growth are drivers. **Characteristics:** People are also extracted from their communities as cheap labour, low livelihood resilience, “rigid livelihood strategies that focus only on one or a few of the options that are available”^*1*^*.* People are very vulnerable to exploitation and shocks (e.g. political, climate events), there are gross inequalities and accumulation of wealth and power. People are replaced through cheaper substitutes or automation (for economic gain), no consideration for cultural impacts. People in poverty are living in a vicious circle, needing to continue extractive practices, as there are no other options or opportunities. Indigenous communities have been displaced, and natural ecosystems are decimated, leading to the emergence of novel diseases (e.g. zoonotic diseases). Toxic chemicals accumulate and magnify, reaping havoc on biodiversity and human health. Heavy impacts on the functioning of ecosystems to provide, for example, healthy air, climate regulation, water purification. Technologies are used for easing the lives of the wealthy and for exploitation purposes. Innovations are only understood as inventing a technology that is for profit, not necessarily related to societal need. Food is unevenly distributed and can contribute to the manifestation of food insecurity (inability to meet adequate food consumption requirements) either at a specific time or all the time, which threatens lives and livelihoods, regardless of the causes, context or duration. **Example:** “Fishing down the food web”^1,2^	**Mindset:** Compartmentalising complex systems. Humans are still separate from nature, in control, we can continue what we are doing if we can cause less damage or limit the damage, FS “delivers food security and nutrition for all in such a way that the economic, social and environmental bases to generate food security and nutrition for future generations are not compromised.“^3^ while pursuing activities that favour and maintain the abundance of only one or a few highly valued key resources. **Characteristics:** People have greater economic opportunities but cannot always invest in their local communities or places where they live, as they still have to move where there are jobs. For some they have gained a level of livelihood resilience, autonomy and collective organisation. However, it is not the same for everyone, some people are still very vulnerable, to potential FS “*disruptions and boom-and-bust dynamics*”^1^ of globalised markets or climate events. Natural habitats that have been fragmented are conserved and protected. Production systems are circular, reducing raw material inputs and closing loops and are no longer dependent on fossil-based sources. The toxicity and accumulation of harmful chemicals in the soils and FS have been remediated and reduced and are within acceptable limits. Smart water systems have been installed that can enhance water use efficiency, particularly to buffer climate impacts. There is a large dependency on technologies and data to provide answers, with some displacement of people in certain economic sectors, but most people can be upskilled again. High yields with low impacts can ensure food and nutritional security for the majority. But there are still many that face hunger. **Example**: “Maine lobster fisheries”^1^	**Mindset**: Being open to the complexity of nature. Humans are a part of nature; a mutualistic relationship exists. All the answers may not be known, but we will learn. “Regenerative systems are high in both flexibility and diversity and entail cultural systems that conserve change by emphasizing responsiveness to environmental cycles and feedback while also valuing ecosystem and food system diversity as outcome”^1^. **Characteristics: **People have greater opportunities to invest in communities and in the places where they are living. They are motivated to look after nature and one another. “Regenerative systems are high in negentropy because livelihood strategies work actively to complement or enhance natural cycles of release and renewal”^*1*^. Building-in livelihood resilience to deal with changing conditions (e.g. political, climate events). Natural habitats and ecosystems are diverse, full of vitality and healthy. They have been given the opportunities (space and care) to coevolve with humans with changing dynamics being embraced and conserved. The natural systems are in balance to evolve, to deal with and protect against bioaccumulation of potential natural toxins or diseases. Technologies are used within a nested knowledge framework supporting greater information and knowledge of how ecosystems function fully and provide feedback, they help people co-exist and enhance the natural world. Technologies are developed in a circular way and potentially open-sourced manner, supporting societal needs. They are partnering with life and upskill people, not causing job losses or inequalities. Food and nutritional security are secured but from a more local and diverse base, diet and relationship to food is in balance with what ecosystems can provide. The food system is nested entirely within a health system conscious of natural feedback loops between humans and nature. There are no disparities, and no one is left behind or hungry. **Example**: Quichua (Kichwa) peoples of Pastaza, Amazonia, Ecuador^4^.

However, developing regenerative pathways towards achieving the goals of RIFS depends first on a change of mindset and worldview, a form of transformative learning (Burns 2015). The first step will be developing the ability to disentangle the indoctrinated narratives, learning and behavioural lock-ins of our current FS and to see them in a new light with a new way of thinking (Iles and Montenegro de Wit 2015). The latter will require new norms and frames of reference to begin understanding the world around us as a living system (Larrick 1997; Burns 2015; Mang et al. 2016; Mehmood et al. 2020; Anderson and Rivera-Ferre 2021). It will also require new imaginaries^8^ to envision and create such a future (Marquardt and Nasiritousi 2022; Schmelzer and Büttner 2024). Anderson and Rivera-Ferre (2021) in their very comprehensive paper on food system narratives identified the importance of framing and narratives of food systems because they can have profound implications for how people perceive or value certain aspects of the food system. They identified that different visions, framings and narratives of our future FSs co-exist, often associated with different, sometimes opposite, mental models. This can ultimately lead to different approaches and solution orientations for the issues within our current food systems. They proposed the use of two narratives to explore and frame the direction and outcomes of future food systems; these are the extractive food systems narrative and the regenerative food systems narrative. For extractive FS as outlined above, the central narrative themes that Anderson and Rivera-Ferre (2021) identified were: “*appropriation and competition.., productivism… modernisation.., consistent with neoliberal economic concepts”*. In contrast, regenerative food system narratives were found to focus on *“activities and imaginaries that can restore or enhance communities and ecosystems (human, social, financial, physical, natural capital) eroded by decades of implementation of extractive narratives”.* There is a tendency today to be fixated on a uniform or global vision of agriculture and food systems. Regenerative FS approaches challenge this. The regenerative narrative recognises the need to have a greater diversity of visions which are rooted in local SESs, being co-developed by the actors involved in these FSs. In this way increasing the long-term FS resilience and adaptability, to embrace dynamics and changes. Visions can help to steer the course of the future, as they represent all the hopes and dreams of a better future, often based on questioning the uncomfortable practices of the present (Dahlberg 1993).

Dahlberg (1993) was one of the first authors to imagine and create a vision for a regenerative food system. They imagined it as a nested system *“.. of agriculture, food systems and societies operating within the larger framework of socio-natural*^9^*systems”* and operating at different scales (e.g. farm, communities, landscapes) and different time horizons (e.g. short term to intergenerational) and within different contexts enabling a closer link with ecosystems to utilise and recognise potential ecological feedback in the systems. Understanding FS in this way implies that these systems have resilience through their regenerative capacities. Regenerative development of the food system is fundamentally place-based, carried out by networks of actors rooted to place, as the relationship of humans to the places where we live, how we experience them, can provide us with a sense of connection and responsibility for the living world of which we are a part (Iles and Montenegro de Wit 2015; Mang et al. 2016). Characteristics of regenerative FS include, but are not limited to: multifunctionality, diverse collaborations and working with nature (e.g. using context specific, local, and traditional ecological knowledge, biomimicry). All of these factors combine in recognition and respect of our differences and interdependencies, while trying to build the capacities to learn and thrive in the complexities of SESs (Dahlberg 1993; Duncan et al. 2020; Anderson and Rivera-Ferre 2021). While the focus of regenerative food systems is local, their nestedness means they do not function in an independent vacuum, but they are part of a bigger system, a global system of different socio-ecological networks or economies interacting and engaging with one another. System nestedness and connections to the “greater whole” must also be taken into consideration when exploring options for regenerating a food system (Sundkvist et al. 2005; Mang et al. 2016; Caniglia and Mayer 2021).

One additional consideration in rethinking and reimagining our food system is autonomy of resources and redistribution of power away from the few (Iles and Montenegro de Wit 2015). This means there is the need to put inclusion “*squarely in focus when analysing long-term pathways for food systems transformation*” (Gaupp et al. 2021).^10^ Thus, as Anderson and Rivera-Ferre (2021) outlined, there is a need to ask targeted questions when assessing transformations in the FS: productivity gains are for whom—what living being or system? Innovations are coming from whom and for what purpose? What are the consequences (intended and unintended, positive, and negative externalities) and capital costs^11^ of such system changes—ultimately, who wins and who pays? Visions and narratives are interrelated and as Anderson and Rivera-Ferre (2021) outline *“if narratives, their associated strategies and their likely outcomes are not made explicit, then there is the risk of creating confusion, particularly at the policy and research levels”.* In the next section, we present our vision for RIFS using four outcome areas to structure and substantiate the narrative.

### The vision for RIFS, using four nested outcome areas

**Fig. 1:**
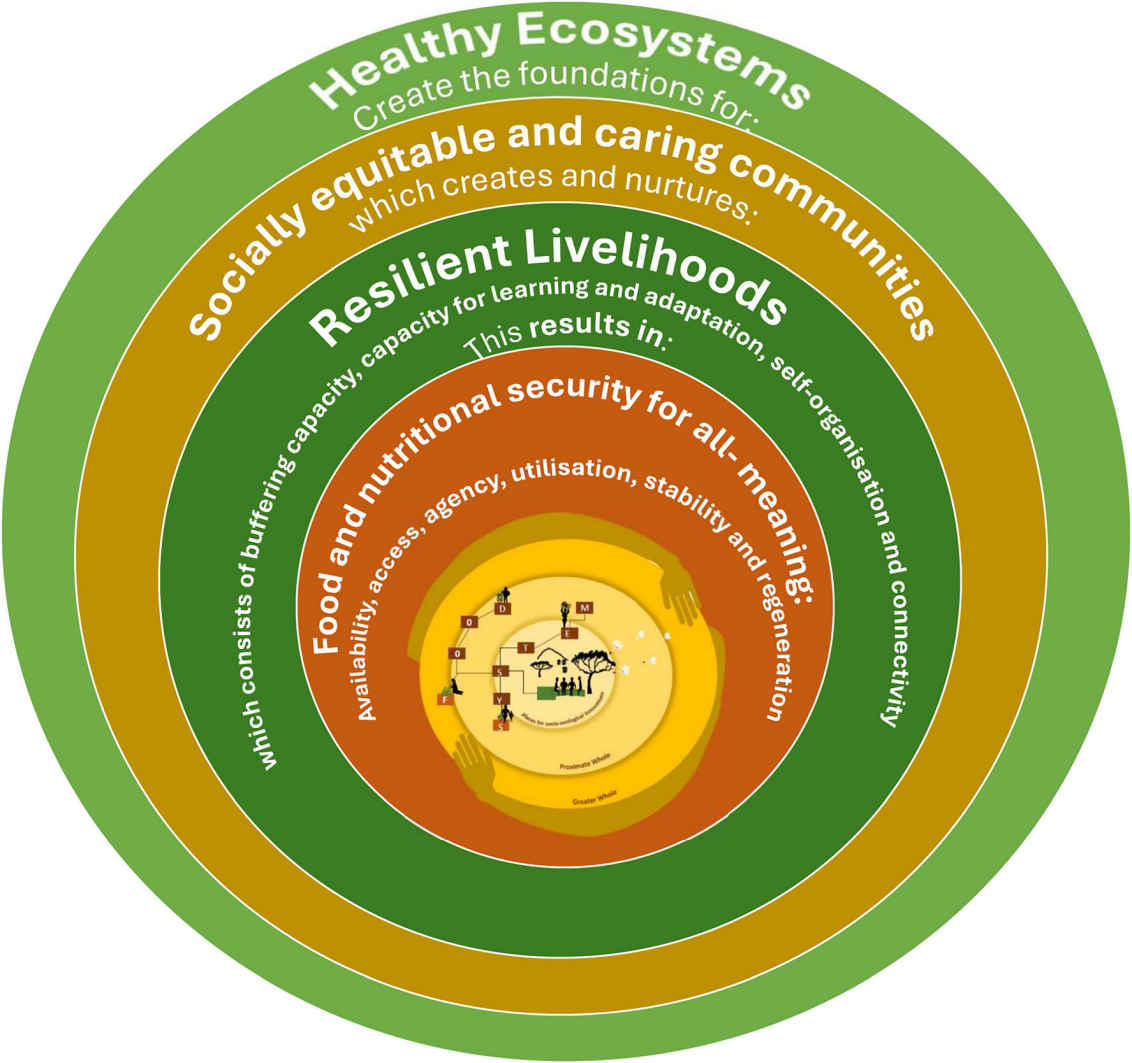
A graphical representation to show the nested nature of a functioning food system as part of a socio-ecological system characterised by four key outcomes which are all interdependent (i.e. the nested rings) to build regenerative capacities and ensuring food and nutritional security for all (adapted from: Burkhard et al. 2008; Ifejika Speranza et al. 2014; Jacobi et al. 2018; UNDP 2018; Du Preez et al. 2020; HLPE 2020; IFPRI 2020)

In this perspective paper, the proposed vision for the future FS is one of regenerative, inclusive food systems (RIFS) nested within greater SES. This is a FS where people work innovatively with nature to foster healthy ecosystems which can create the foundations for socially equitable and caring communities, and which in turn creates and nurtures resilient livelihoods, ultimately leading to food and nutritional security for all. In this fair and just transformation, no being is left behind. These outcomes require the place-based strategies and regenerative actions taking place locally, while not forgetting the connections to the greater whole or bigger system. Figure 1 visualises the four outcome areas and the nested nature of a functioning food system.

Taking the outermost ring which creates the foundations for the other outcomes, we understand healthy ecosystems using the concept of “Ecosystem health”. A healthy ecosystem is one that has the ability to maintain its structure (organisation) and function (vigour), especially in relation to the ecosystem services it provides to humans and non-humans alike, over time, regardless of external stresses (resilience) (Costanza et al. 1999; Burkhard et al. 2008).

The second ring refers to the creation of the social conditions, which facilitates and enables the other three outcomes. For socially equitable and caring communities we use the LNOB, “Leave no one behind” framework to understand the situation we want to move away from. This was developed under the UNDP, which identifies that *“people get left behind when they lack the choices and opportunities to participate in and have a proportionate share in the benefit and burdens from development progress”.* All persons living in extreme poverty can thus, be considered as ‘left behind’, as can those who endure disadvantages or deprivations, five key factors play a role in LNOB: discrimination, shocks and fragility, geography, socio-economic status and governance (UNDP 2018), see Table S1.6. In RIFS, “no being is left behind”, including also nature. When describing caring communities, there is a need to traverse diverse types of literature and disciplines ranging from: palliative care, to feminist political economy, to care farming, to education, among others (Carson 2017; Wegleitner and Schuchter 2018; Hassink et al. 2020; Barca et al. 2023). The action of “care” is defined as “the ability to being responsible for, attending to, being concerned for or about, and paying watchful attention to the object of care” (Hassink et al. 2020). Therefore, a caring community can be recognised as one which shares a mutualistic understanding, marked by respect, integrity and caring for each other, affirming the inherent dignity of individuals and the collective as a whole, where expression and voice can be raised within the bounds of courtesy, sensitivity and respect (Carson 2017). Furthermore, such communities “*sees plants and animals as community members, rather than commodities, and that wishes to move from an attitude of control towards one of partnership and respect*” (Hassink et al. 2020), extending the focus from the domestic realm to that of the land and non-human environment. In this way caring communities challenge the culture of individualism, undoing the geo-supremacy perspective, while still working within states (Barca et al. 2023).

The third and fourth rings refer to the key functioning roles that the FS should play in society. For the third ring, we use the concepts of resilient livelihoods, defined by Ifejika Speranza et al. (2014) as “*the capacity of livelihoods to cushion stresses and disturbances while maintaining or improving essential properties and functions…. A livelihood is thus resilient if it can maintain its key functions (food, income, insurance, poverty reduction, *etc*.) and absorb the impacts of disturbances without causing major declines in production and well-being. Livelihood resilience thus, depends on how well a livelihood functions, on actors’ capacity and agency, and on the social, institutional and natural conditions*”. For the most internal ring, food and nutritional security, there are six crucial components to determine the state of food and nutrition security. These are availability (production, distribution, exchange); access (physical, financial, social, preference); utilisation (nutrition, safety); stability; sustainability and agency (HLPE 2020).

All four outcomes of RIFS are dynamic and influence each other through diverse interactions among multiple contextual factors—including, socio-cultural, economic, political and environmental conditions, the extent to which will determine the ability of the system to retain its regenerative capacities (Loring 2021). More in-depth descriptions of the outcome areas and example characteristics are provided in supplementary material S1. While reimagining an alternative future FS is the first step towards understanding how to regenerate the current one, the next step is to have a set of guiding principles to help navigate the complex processes involved in trying to achieve RIFS.

## Navigating complexity: Guiding principles, values and frameworks

### General methodological considerations

The authors of this paper comprise of food system development practitioners, regenerative practitioners, as well as action researchers and researchers from different disciplines and countries. In the process of trying to understand RIFS, how to develop them and to measure their success, no practitioner guidelines or approaches existed for supporting the development of RIFS in practice. Therefore, we took an approach similar to Cummings et al. (2023) and used a “snowball sampling” of literature (peer-reviewed and grey) to identify relevant concepts and frameworks. As a result, in this paper we refer to a broad spectrum of scientific fields, for example: regenerative agriculture, agroecology, regenerative development, socio-ecological systems, biomimicry, circular economy, transformative education, just transitions, living system theory, food system resilience and food sovereignty. Similarly to Cummings et al. (2023) our approach could also be described as a “methodological bricolage”, which has three characteristics: making do, utilising the resources at hand and combining resources for new purposes (Baker and Nelson 2005; Pratt et al. 2022).

First, as regenerative inclusive food systems are still an emerging concept we have to “cobble together” a coherent argument, for a vision of RIFS and what key outcomes this entails, as well as what principles could be useful to steer the process. We did this through our own experiences as action researchers and through further engagement with the literature on regenerative development and other relevant research fields to scientifically underpin it. In this paper we lay out our arguments in a manner to build trust in our approach and to convince the reader of both the practical value and the scientific underpinning of the RIFS principles. Second, we tried to utilise as best as possible all the resources available. Furthermore, we collaborated with the diverse team involved in the REFOOTURE project to sounding board and co-develop the RIFS principles and outcomes (Supplementary material S2). Thirdly, we combined existing resources to help understand the complexity of regenerative development and food systems, with the aim of it being useful for on the ground practitioners. Through reading the available literature on regenerative development and synergistic or complementary approaches and movements relating to food systems, it was found that many of the approaches reviewed used principles or values to provide direction and guidance for change processes in very complex systems (Supplementary material S3). Therefore, based on the four outcomes for RIFS we wanted to understand what principles could be used to help steer the processes to support the emergence of RIFS. We further outline the method employed in the succeeding sections.

### Deriving RIFS principles: Approach taken

Principles can serve as a compass to navigate through unchartered processes, experiences, uncertainties and turbulences which emerge from complex dynamics involved in transforming a FS. They also provide the lenses through which to identify potential opportunities and challenges. In particularly *“overarching principles can provide the big-picture and general guidance”* (Patton 2018). Principles also can be used to provide direction to activities aiming to achieve the regenerative goals of a particular place-based project (Mang and Reed 2020). Overarching principles can encourage us to ask what is really meant by RIFS and to constantly check what the principles mean in practice and how we can translate them into meaningful actions and behaviour. The principles encourage discussion and experimentation. They ask us to shape and adapt our vision, but they do not prescribe the vision (see section “Putting Theory into Practice).

Many of the regenerative development approaches and synergistic or complementary FS approaches and movements reviewed (Supplementary material S3) have been triggered by people’s need to imagine alternative versions of our FS and to disrupt our complete transition to fully industrialised, globalised and automated FS. This desire to reconfigure and change direction is increasing in momentum and can be seen by the growing discussions around, for example, regenerative agriculture, circular agriculture and agroecology. Regenerative agriculture, in general refers to on-farm level and while there are some papers that argue for a broader scope and definition (Sands et al. 2023), there is an ever-increasing narrative to focus regenerative agriculture on soils (Giller et al. 2021; Schreefel et al. 2020). In regard to circular agriculture, while again offering many practices that align with regenerative FS, the focus is more on socio-technical solution sets, than truly working with nature. Agroecology as a movement and practice shares many key defining features with regenerative food systems, from sourcing ecological wisdom from indigenous people, to seeking to understand the contexts of the larger food system, to creating solidarity in the food system (Dahlberg 1993, 1994; Duncan et al. 2020; Sands et al. 2023). While all three, especially agroecology, have similarities, they also differ in how they are currently being implemented. They are predominantly implemented using a sustainable development lens (HLPE 2019; Wezel et al. 2014; Gliessman 2017; FAO 2018a, b), where the focus is on minimising negative impact to acceptable levels (i.e. “doing less harm”). This is different from the regenerative lens, which explicitly aims at generating positive impact and giving back (Table 1). Therefore, we build here on the existing movements’ learning and transpose their valuable insights through the regenerative lens to understand better how such positive impacts can be encouraged and created. There are several common key characteristics of these diverse approaches. The associated principles that we assessed are provided in Table S3.1. While not an exhaustive list, we reached a point where almost no new universal characteristics emerged. This was the point at which we considered the sample to be sufficiently saturated. These universal characteristics were related to the promotion of:

- place-based approaches creating a sense of place through reconnecting humans with nature and with place.
- a collective purpose—using a common purpose to build mutualistic relationships.
- holistic systems thinking—working with a bigger picture and building connections to something bigger, promoting diversity of people, knowledge of place.
- accountability and stewardship for innovations—promote accountable innovations that are value-adding^12^;
- mutualistic connections and relationships (strengthened through a common purpose).
- autonomy and responsibility of decisions and resources by communities and local people.
- adaptive and collective learning—regenerative capacities.
- designing for long term (intergenerational).

**Fig. 2:**
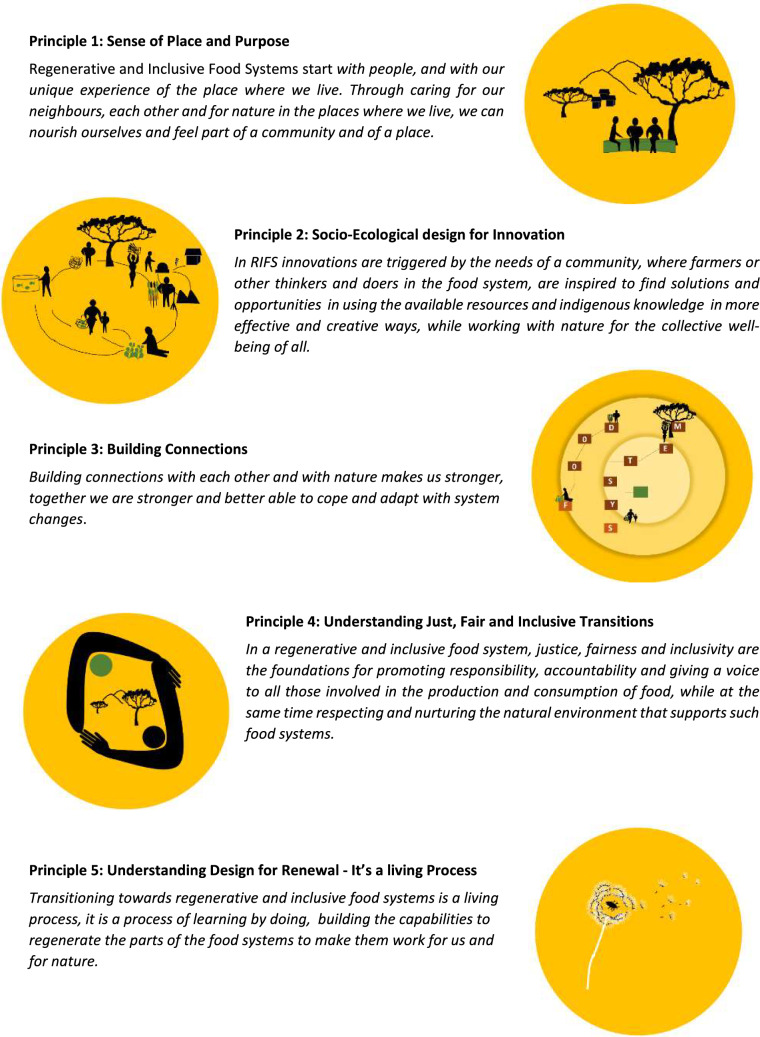
All 5 RIFS principles and their summarised descriptions, as well as the first drafted graphical representation of the principles

Building on the valuable lessons learnt by the developers and promotors of these related principles, this list was used as the basis for exploring, structuring and sense-making^13^ (Weick et al. 2005) of the different interdisciplinary conversations and activities carried out within the REFOOTURE project in order to determine key principles that could provide direction to the processes of moving towards RIFS. Due to Covid restrictions, discussions and workshops had to be held online. These include three round table dialogues between living lab members in the East African countries of Ethiopia, Kenya and Uganda,^14^ as well as several bimonthly project meetings, where varying aspects about the different regional food system were shared and discussed. Many times, the discussions involved an interdisciplinary and international team of researchers, however on several occasions, different community members active with the associated living labs, came to share their knowledge also with the research team. Using these discussions and the literature, as the resources available to us, five principles were identified. A set of five principles was seen as a pragmatic and manageable number to work with in the field. The symbols were produced to support the diversity of thinkers in the group (e.g. more visual leaners) and to support potential communication on the ground. A sound boarding workshop was used to refine and test the resonance of the principles within the REFOOTURE team. The five RIFS principles are outlined in Fig. 2. While we have developed these principles in the context of the REFOOTURE project in East Africa, Table 2 shows examples of real-life cases where the principles can be identified in practice around the world, showing that they can be applied and observed in differing contexts. In the succeeding sections, we outline what the principles should ideally encompass underpinning them scientifically with relevant literature and providing examples of where they can be seen in practice. Furthermore, we indicate when there are links between the RIFS principles.

**Table 2:** A small selection of projects and initiatives from around the world that show examples of the five RIFS principles in action. Bold font indicates that these are the more dominant principle. Many of the examples presented in this table were found using the website “Seeds of good Anthropocene”: https://goodanthropocenes.net/, which provides inspiration visions and stories for a”just, prosperous, and ecologically diverse world”. Sources for Makapads: https://goodanthropocenes.net/?s=makapads; https://www.youtube.com/watch?v=jkhVmKkphN8/; https://www.youtube.com/watch?v=jkhVmKkphN8. Sources for Raddis: https://www.raddiscotton.com/. Sources for Hudson Rivers flows: https://www.hudsonriverflows.com/about. Sources for Sitio Vale das Cabras: https://goodanthropocenes.net/view-seed/?seed_id=168; https://sitiovaledascabras.com.br/. Sources for Satoyama Initiative: https://goodanthropocenes.net/tribal-parks-2/. https://satoyamainitiative.org/concept/satoyama-initiative/

Examples	Country	Principle 1: Sense of place and purpose	Principle 2: Socio-ecological design for innovation	Principle 3: Building connections	Principle 4: Just fair and inclusive transitions	Principle 5: Design for renewal, it’s a living process
Makapads	Uganda	Linked to the place through the non-extractive use of natural resources (Papyrus) Enhancing and empowering the community through living wages	**Uses indigenous decentralised fabrication process while supporting local ecosystem (Papyrus)** **Chemical-free and biodegradable** **Using solar power for sterilisation**	Connects many experts and people from disadvantaged communities	**All girls have the right to education and should not have to miss school due to lack of access to sanitary protection** **Employs refugees and vulnerable people, especially women**	**Aiming to improve production, it extended into learning how to improve the sanitary system of schools, refugee camps due to the start of using the pads in first place**
Raddis	India	Linked to the place through the non-extractive use of natural resources **Enhancing and empowering the community through living wages and regenerating nature and removing the use of harmful chemicals in the production of cotton**	Uses chemical-free cultivation and processing of cotton Intercropping food and fibre to establish food and fibre landscapes	**Connect to communities to empower them** **Connecting with NGOs to support community and agronomic developments** **Connect to businesses along the supply chains to build a regenerative value network**	Reconfiguring the business model of cotton sourcing—ensuring livelihood of farmers Advocating for farmers’ right to diversify their production without chemicals Supporting rural women’s rights	Experimenting with agronomic practices Co-learning with the community along the cotton value network
Hudson River flows	America	Linked to the potential of the Hudson River, as a connector of people to place, focusing on empowering communities in the river valley involved in the food system	Intertwining healthy habitats and communities using agroecological practices Creating business platforms for local food producers, “giving back” to local producers	Facilitating and encouraging FS stakeholders to meet and gather to share experiences and understandings Connecting to traditional wisdoms (grounded in science)	**Building capacities around: Food and labour justice, Farmland access, food distribution** **Ensuring equitable finance for SMEs** **Different business and ownership models (collaboration not consolidation)** **Advocating for policy making to ensure their region’s regeneration**	Making land affordable for coming generations Piloting options that can support farmers trying to regenerate the region
Sitio Vale das Cabras	Brazil	Community regenerated 2 ha in the Campinas Environmental Protected Area to become a good practice farming centre for socio-environmental transformation They bring together local organic products in a direct way to the consumer/associate	**They use socio-environmental innovation for water management, agroforestry bioconstruction environmental education and conscious food** **They use traditional knowledge and techniques combined with current technologies for building** **They produce solar energy and “give back” to the community**	They use a multidisciplinary team of environmental managers and educators to run the farm They connect communities, nature and food working as a CSA farm	Have a cooperative for generating renewable energy to provide community access to renewable energy sources. It is a model they plan to scale	Provide environmental education to school children, showing what can be done while producing in a more regenerative manner Building the houses required collaboration and co-creation provide learnings and mutual experiences that value the human relationship and generate a new awareness for social transformation
Satoyama Initiative	Japan /international	**Promotes societies in harmony with nature, positive human-nature relationships** **Management at the landscape level can help to secure ecosystem services and conserve biodiversity, and therefore support well-being for humans and nature**	As an international platform supports and promotes: Resource use within the carrying capacity and resilience of the environment Cyclic use of natural resources Values indigenous traditions and culture	**Connecting people to people and people to nature** **Multi-stakeholder participation and collaboration in the multi-functional management of natural resources and ecosystem services**	Exploring new forms of co-management systems or evolving frameworks of “commons” while respecting traditional communal land tenure	Integrating traditional ecological knowledge and modern science to promote innovations

### Principle 1: Sense of place and purpose

Regenerative, Inclusive Food Systems (RIFS) start with people and their connection to place, their sense of being in relationship with something alive and coherent. The idea of place enables people to develop a concrete understanding of the role they must play if they are to sustain mutualistic relationships with nature. This understanding is tangible and is often expressed through metaphors of family and membership. RIFS facilitate this reconnection and relationship to nature and land (e.g. Satoyama Initiative, Table 2), to the uniqueness and livingness of the places where people live (e.g. Hudson River flow, Table 2), returning them back to their core position at the centre of human life and society. In this way, they reclaim their position as a source of shared meaning and caring that can enable people to have a common cause with one another and with nature (Fullerton 2015; Mang et al. 2016; Gibbons 2020). Determining a collective sense of purpose or vocation related to a place, that we call home, can be a powerful and unifying social force, enlivening local culture, educational practices, livelihoods, and governing practices. It can encourage and enable people to work together, regardless of difference, to switch from extractors and “owners” of nature’s capital to being part of a system that coevolves (e.g. Sitio Vale das Cabras, Table 2). In this way, humans become increasingly able to care for and enrich nature and the socio-ecological systems (SES) within which we are nested (Mehmood et al. 2020).

Caring informs action. Indeed, according to the MA, (2005), a common finding from community assessments indicated that local communities understand that caring for their local ecosystems, protecting the diversity of species and food sources was a form of risk management, of creating “saving banks” and “buffers” to create greater resilience during adverse times. At a functional level, proximity to those who produce our food, along with understanding how it is produced, promotes greater awareness of ecological feedback and how to adjust and adapt the local SESs to ensure food system resilience (Dahlberg 1993, 1994; Sundkvist et al. 2005). Such knowledge is amplified by a sense of collective purpose, ownership and caring, which helps to overcome barriers to the collaboration needed to carry out concrete, complex, large-scale, and place-specific actions. As communities see the positive impacts of such efforts, they initiate a virtuous cycle of caring, accompanied by increasingly ambitious efforts to enhance the vitality and viability of a place (Mang et al. 2016; Mang and Reed 2020). However, the effort of this care should not be inequitably distributed into more gendered roles, therefore, feminist perspectives and approaches are also required to underpin and steer regenerative place-based actions (Manuel et al. 2024).

Food systems are nested systems and therefore do not operate within an independent vacuum, but within regional, national and global economies. Therefore, the aim of place-based and regenerative approaches underlying RIFS is not to solely focus on local FS, but to promote regeneration of FS at all scales, using place as a leverage point for triggering these changes (Sundkvist et al. 2005; Mang et al. 2016). This comes from the premise that when one part of the basic level of a living system is enabled and encouraged to thrive, there is the potential to create greater positive and cumulative outcomes that can far exceed initial expectations and ripple through to other scales of the living system (Larrick 1997; Mang et al. 2016). Indeed Mehmood et al. (2020) described place-based approaches where “*places are seen as sites of incubation and spatial networking that also inspire social initiatives and innovations… establishing links between different spatial scales and communities (upscaling) and building new linkages”* (principle 3). In such places, socio-ecological innovations (principle 2) can create the confluence between people and place, creating self-efficacy, local values and a deviation from undesirable practices that can lead to greater regeneration of community and place. The spatial anchorage or entry point for place-based approaches can be diverse, based on for example, specific pedoclimatic conditions (e.g. Satoyama region, Japan); the use of locally available natural resources (e.g. Makapads, Uganda), including plant varieties or animal breeds (e.g. food fibre landscapes, Raddis); local diet preferences, knowledge, or other cultural traditions (e.g. ecological buildings, Sitio Vale das Cabras); or specific institutions (e.g. Satoyama). However, in FS, where the spatial distances between actors and activities are much greater, there is still a need to enhance communication between the different actors in the food system across the various places. This will allow important knowledge on potential ecological feedback to be relayed though effective channels of governance and institutions (e.g. polycentric governance and subsidiarity), principle 4 (Sundkvist et al. 2005; Iles and Montenegro de Wit 2015).

Therefore, the fundamental premise behind this principle is that in RIFS, placed-based approaches, which can create ripple effects into the larger FS, should be encouraged to help create more caring for nature and community, laying the foundation for other regenerative activities.

### Principle 2: Socio-ecological design for innovation

For RIFS, ecological design is fundamental to transitioning our social systems to a greater reliance on renewable resources through recycling, reusing and dematerialisation (CGR 2021, 2022). Furthermore, such designs need to provide greater opportunities to be fossil-free (e.g. Makapads, Table 3). We need to be able to mimic the fundamental designs, networks and structures of nature, while at the same time enhance those diverse natural systems by becoming more aware of ecological feedback (Benyus 2009; Shu-Yang et al. 2004; Sundkvist et al. 2005). In ecological economic theory, according to Berkes and Folke (1992), without natural capital, there would be no foundation for humans to develop human-made capital. This means we need to be more conscious of how we care for the Earth’s natural capital. Therefore, at the design phase of any innovation we need to learn to think mutualistically about the potential social (including economic) and ecological consequences of our ideas and food system activities (e.g. Sitio Vale das Cabras, Table 2). Loring (2021) identified that regenerative food systems cannot be created by merely adapting the current systems. For RIFS to materialise, a paradigm shift is required that employs alternative strategies to restore and enhance the five capitals of SESs, while compensating and healing past harms created through extractive and exploitative practices (e.g. Raddis, Table 3).

It is largely recognised that transition through system innovations requires a diverse set of changes, initiated, implemented and advocated by a diverse set of actors, particularly innovations with the aim of enhancing SESs. These cannot be set in motion purely in a top-down manner, simply because the complexity is so great that individual decision makers do not have the overview or the means (e.g. financial, knowledge) to drive them (principle 4). However, top-down support from institutions (e.g. Satoyama Initiative, Table 2) and governments is still pivotal in creating an enabling environment for innovation, particularly through regulatory interventions, funding mechanisms and their roles to ensure a more equitable distribution of benefits (Baker and Mehmood 2015). Therefore, it needs to be a combined and concerted effort of all actors^15^ (Loorbach et al. 2017; Kemp et al. 2020; Loring 2021; Loorbach 2022). With this in mind, we look to the concepts of social innovations^16^ which seek not only to introduce new types of products or services not yet provided by the market but also seek to introduce more novel ways of using existing products and knowledge (i.e. reconfigure or regenerating aspects that do not currently bring added value to the majority). Such social innovation encourages and motivates the role of human agency to catalyse a change in existing attitudes,^17^ reconfiguring social networks and governance and to improve the collective well-being of the community (e.g. Hudson River flows) (Secco et al. 2020; Baselice et al. 2021). Social innovations are also grounded in place (Baker and Mehmood 2015), these coupled with regenerative action can create a new synergistic, innovative agency and a more conscious way of acting (Mehmood et al. 2020). One concrete example of regenerative action in the food system is the holistic approach promoted by agroecological practices (Dahlberg 1993, 1994).

When it comes to the use of socio-technical innovations (e.g. machinery or digital technologies) within the food systems, we also need to ask important questions around the issues relating to the autonomy of the resource base, data security, rights and access (Gkisakis and Konstantinos 2020; Van der Ploeg 2021). It is important to ask: what are the technologies being promoted?, by whom?, who has ownership and rights?, who benefits and what is the relationship between such technologies and local innovations and knowledge? (Nyéléni 2019). Finding tailored solutions to manage the diversity of needs across the food system should also be reflected in the types of tools that are developed. In many cases this should be a co-designed process. Indeed, promoting the collective mobilisation of farmers and other relevant stakeholders to build knowledge networks and coevolve useful ideas, which are respectful of place-based contexts (Principles 3, 4, 5). This in turn, could mean that the current paradigm or agricultural innovations being a “one-type-fits all” or “top-down” solutions designed by the agroindustry will become redundant, or at least less dominant. Thus, potentially giving farmers the rights to their own designs (e.g. Sitio Vale das Cabras, Table 2) and their own data (Gkisakis and Konstantinos 2020; Salembier et al. 2020).

Therefore, the fundamental premise behind this principle is that merging social innovations with the conceptual thinking and framing of ecological design can create new, more satisfactory ways of giving people and nature a place within RIFS. Such innovative ways of production start when people focus on the potential around them (Principle 1), helping them to identify more powerful opportunities which are generally not realised when people work from a problem–solution orientation (Mang et al. 2016). This is because the “thought silos” are removed, and a fuller living system can be observed. This mindset of production in RIFS can lead to encouraging healthy ecosystems, equal and caring communities, while also promoting livelihood resilience of the local communities, which in turn ensures food and nutritional security.

### Principle 3: Building connections

RIFS are living systems nested within larger living systems (e.g. watersheds, biomes, communities, families). The longevity, vitality and fitness of regenerative food systems are tied directly to their beneficial integration into a larger system (e.g. Hudson River flows). The quality and strength of these connections among people and nature are critical to the transition towards a regenerative, inclusive food system (Dahlberg 1993; Mang et al. 2016). Connectivity is established in diverse ways, including physical relationships (e.g. to places along rivers, between roads and railways), digital relationships (e.g. internet, information technologies), and social relationships (e.g. family, neighbourhood, community) (de Steenhuijsen Piters et al. 2021; Secco et al. 2020).

People-to-people connections (e.g. Table 2) build social capital, which *“describes the way in which people form connections through relationships and networks built on trust and reciprocity in order to enhance cooperation, collective action and resilience”* (Niles et al. 2021). There are two distinct types of social capital. Structural social capital and cognitive social capital. The latter refers to having shared norms, values, attitudes, trust and beliefs. By contrast, structural social capital refers to the sharing and dissemination of information, and the collective action and decision-making governed by specific rules and guidelines, which are facilitated through established roles, social networks, and other social structures (Grootaert and Van Bastelar 2002). Structural and cognitive social capital are established through three recognised activities: bonding (e.g. relationships within social groups), bridging (e.g. building relationships among different social groups) and linking (e.g. networking between individuals and groups with differing social positions) (Kizos et al. 2014). The creation of strong social capital can also encourage a collective sense of place and purpose (Principle 1). Underpinning and establishing social capital using epistemic justice concepts ensures a greater cohesion between cognitive social capital and structural social capital (e.g. useful knowledge will be shared with those that may hold differing beliefs or that may belong to more marginalised groups) (see Principle 4).

Cultural capital can be described as “*the aptitude or inclination of a group or society to behave in a certain way, which underlies human and social capital and describes the potential of a group or society”* (Cochrane 2006). This aptitude has been shaped by the geographical space that a cultural group co-inhabits with nature (Berkes and Folke 1992). Thus, cultural capital informs the guiding rules and interactions over time between communities and their natural, historical and social environments. It also shapes the means with which societies deal with their natural environments and how they have derived their identities from their local surroundings (Cochrane 2006; Kassam 2009; Reyes-García, 2015). Social and spiritual relationships often have ecological foundations, with the practical manifestations of cultural practices and activities, in turn, having consequences for local ecosystems (Kassam 2009).

Social and cultural capital are important forms of connectivity and are vital for RIFS, not only because these determine how natural capital will be converted into other forms of capital, e.g. economic, or physical (man-made) capital (Berkes and Folke 1992; Kizos et al. 2014), but also how these forms of capital will be shared and used. Social capital is associated with decreased risk of hunger (Martin et al. 2004), among other benefits, and it is increasingly being recognised that a lack of social capital can have consequences for health, poverty, and inequality (Chetty et al. 2022).

Therefore, the fundamental premise behind this principle is that in RIFS, social and cultural capital should be activated or developed to better ensure synergistic relationships with nature, social equity, resilient livelihoods, and food and nutritional security for all.

### Principle 4: Fair, just and inclusive transitions

In its current form there exists many forms of injustices within the FS; this has led to many underlying inequalities and power disparities, as well as persistent and wicked problems in the FS, with the dynamics of exclusion being embedded throughout (Hebinck et al. 2021). The current power concentrations in the global market and the structures enabling them, have been identified as a major cause of inertia and lock-ins found within the current food systems (McKeon 2021; Tschersich and Kok 2022). Therefore, for RIFS to emerge we need to create a fair, just and inclusive transition, one that “levels the playing field” for all stakeholders and can create win–win-wins for those with power, those with limited or restricted power and for nature. Indeed, “*reshaping food systems to be inclusive of poor and vulnerable people is a moral imperative*” (IFPRI 2020). It is fundamental that any transition which aims to reconfigure the food system must be grounded with social and ecological considerations (Principle 1–3), that work together in ways that are regenerative, enhancing a balanced set of ecosystem services and providing food and nutritional security for generations to come (Berkum et al. 2018).

The concept of “just transition” has its roots in the labour and environmental justice movements which focused on improving regulations for environmental and working condition and which has now also been adopted by the climate and ecological justice movements (Tschersich and Kok 2022). For system change to occur motivation, commitment and critical mass—a support base large enough to create the momentum for change is required. Particularly important are the commitments to actions and it is crucial that these are agreed upon, implemented and advocated by as many stakeholders as possible, as this is what creates change (Berkum et al. 2018). However, it is also imperative that in any just transition process there is a need to have an explicit awareness of the potential creation of adverse social impacts or the reinforcing of structural injustices that do not work for everyone (Connix et al. 2022; Tschersich and Kok 2022) or for nature. De Bruin et al. (2024) recognised, in their analysis of political philosophy and politics of justice in food and agricultural systems, it is critical to be reflexive to engage with the underlying values, ideas and causes of social inequalities. This needs to be done in an inclusive manner, to enable those to be recognised as “subjects of justice”, who have a particular role in the food system, both as producers and consumers. They also recognised several groups that need greater considerations in a just transition, these are marginalised or disadvantaged people, indigenous communities, those that have experienced negative effects of FS, future generations, and non-humans*.* Just transitions in the FS consist of approaches aiming to disrupt and reconfigure embedded structural and societal silos considering stakeholders’ rights, scales and time while simultaneously supporting the rights of nature (Borràs 2016; Coninx et al. 2022;18 Putzer et al. 2022; Putzer et al. 2025).

For the diversity of stakeholders involved, there needs to be considerations of four types of justice conceptualisations (Coninx et al. 2022) including: (1) *distributive justice*—ensuring a socially equitable distribution of benefits; (2) *procedural justice*—ensuring every voice is heard equally and fairly; (3) *recognition justice-* providing dignity and respect equally to all active in the FS, particularly those groups who are recognised as have had or still suffer injustices within the FS; and (4) *restorative justice- which is defined as “a flexible, participatory and problem-solving response to criminal behaviour which can provide a complementary or an alternative path to justice. It can improve access to justice, particularly for victims of crime and vulnerable and marginalized populations, including in transitional justice contexts”* (UNODC 2020).

Transitions in a FS will occur at different scales, while RIFS promotes working with the transformative potential of place (P1), due to the nested nature of these systems, efforts to transform a food system in one region or country may have negative social impacts elsewhere, which may also in turn create positive feedback for these places. Therefore, these potential issues in the bigger global food system still need to be, acknowledged, anticipated, prevented or addressed (Coninx et al. 2022). Another consideration for a fair, just and inclusive FS is the consideration of time, of “distant voices”, the future generations who will be affected by decisions made today but are unable to participate in the governance process of the FS transitions (De Bruin et al. 2024; Tribaldos and Kortetmäki 2022). Furthermore, while the considerations of (human) stakeholders need to be held into account in such a transition, the rights of nature (non-human) need also to be considered. This means acknowledging the natural world’s rights to exist, persist and maintain its vital cycles (e.g. the right to flow for rivers and the right to exist for ecosystems) (Borràs 2016; Putzer et al. 2022; Putzer et al. 2025). Putzer et al. (2025), in their work inventorying rights of nature initiatives with over 495 initiatives spread globally across 40 countries and territories, show this is a quickly evolving movement with many legal activities focused on providing rights of nature.^19^

To support the uptake and operationalisation of these different forms of justice to enable RIFS to emerge, will also require a reconfiguration of governance, as FS are dynamic SESs, constantly changing and evolving. This means that the governance^20^ of such systems also needs to be dynamic (Tribaldos and Kortetmäki 2022). Berkum et al. (2018) identified that “*one of the principal causes of a food system’s failure to evolve in desired directions is its governance”.* Establishing policies which focus on controlling one to a few ecological aspects or processes will not facilitate an ecosystem’s capacity to adapt and change and instead will lead to creating more fragile and vulnerable SESs. This is because SESs are highly complex, characterised by cross-scale spatio-temporal interactions. Decisions that are taken today in a specific place and time can also affect people today or in the future and elsewhere (Petrosillo et al. 2015; Tschersich and Kok 2022). This will make planning and governance of these systems challenging. Therefore, enabling a just, fair and inclusive food transition will require “*adaptive, reflexive and pluriform governance efforts that confront fundamental inequalities and redirect vested power relations that stabilize status-quo configurations”* (Tschersich and Kok 2022). Such governance efforts need to build from the knowledge and learning gained from governing the commons (Ostrom 2010). The fundamental theory for commons management is “polycentricity”, this is defined as a complex form of governance, using principles of subsidiarity, with multiple governing authorities and decision-making centres at different scales managing the SES, rather than one centralised body. While each decision-making centre has semi-autonomous independence to create their own norms and rules within a specific scale or domain, they still need to take into account the other decision-making centres in a cooperative manner with established conflict resolution mechanisms (Iles and Montenegro de Wit 2015; Gatto 2022; Kellner et al. 2024). This multilevel configuration means that governance arrangements operating in polycentric manner are able to balance decision-making between centralised and fully decentralised, as well as community-based governance (Kellner et al. 2024). Furthermore, if implemented successfully it facilitates the interaction between public and private sectors with civil society. The result being a win for all (Gatto 2022). Gatto, (2022) provided two examples, using water governance from two different regions, Latin America and Italy to illustrate how reflexive and pluriform governance can work in practice, redistributing power to enable more participatory approaches in decision-making for communities, indigenous populations and other various stakeholders dependent on the management of a particular water source. Furthermore, the case of South America highlighted the ability of such pluriform of governance to recognise the rights of all living creatures as part of a living ecosystem, those present now and also the generations (human and non-human) to come. In Table 2, many of the initiatives also focus on different elements of justice (e.g. land rights, labour rights), new modes of conducting business (e.g. collaboration not consolidation) based on cooperative models and models which impower producers, as well as commons management.

Therefore, the fundamental premise behind this principle is that the vision of RIFS can strive for an inclusive just and fair foundation, learning and building from concrete examples and initiatives that exist already, providing greater opportunities to create the enabling conditions for ensuring synergistic relationships with nature, social equity, resilient livelihoods, and food and nutritional security for all.

### Principle 5: Design for renewal (it’s a living process)

The backbone of a regenerative, inclusive food system that delivers the desired outcomes of healthy ecosystems, equitable and caring communities, resilient livelihoods, and food for all, is resilient SES (Ifejika Speranza et al. 2014; Anderson and Rivera-Ferre 2021). However, resilience is not an end state, and it may not always be intrinsically positive^21^ (Tanner et al. 2015). Therefore, resilience needs to be understood in relation to specific contexts, social values, and norms. We must ask important questions such as, resilience of what type, and for whom? (Cretney 2014; Ifejika Speranza et al. 2014; Tanner et al. 2015; Jacobi et al. 2018). For SES, social resilience is a dynamic concept that not only refers to peoples’ capacity to buffer system changes and shocks (e.g. political upheavals, climate change), but also refers to their capacity for learning and self-organisation (Ifejika Speranza et al. 2014; Jacobi et al. 2018). Ifejika Speranza et al. (2014) defined social resilience as *“the capacity of actors to access [livelihood]*^22^*capitals in order to—not only cope with and adjust to adverse conditions (i.e. reactive capacity)—but also search for and create options (i.e. proactive capacity), and thus develop increased competence (i.e. positive outcomes)”.* Furthermore, they contextualised social resilience as livelihood resilience, determining that the ability of someone and their livelihood to cope with change and to recover characterises their level of resilience (Ifejika Speranza et al. 2014).

The more we manage our food systems for stability and uniformity, the greater the chances that we reduce their ability to adapt and to build the necessary capacities for change. For food systems to be regenerative they need to “conserve change”, to ensure that future cycles of regeneration are possible. This conservation includes everything from maintaining the diversity of ecosystems and cultural knowledge, to the diversity of livelihood strategies (Loring 2021). RIFS are living systems; they are dynamic and in a continuous state of change, which “*requires us to treat change as a source of creativity*” (Mang et al. 2016; Mang and Reed 2020; Loring 2021) and to be conscious of its sovereignty, intentionally building, nurturing and maintaining relationships between people, institutions, technologies and ecosystems across and between nested systems (Iles and Montenegro de Wit 2015). However, to engage with the dynamic nature of food systems will require a change of mindset and a different way of thinking and approaching how we explore and understand them (Mang et al. 2016; Anderson and Rivera-Ferre 2021; Loring 2021), particularly, if we want a fair and just food system transition (principle 4). It is clear therefore, that we need to undergo a type of transformative learning^23^ (Mehmood et al. 2020).

In regenerative systems, observation, experimentation, and the ability to adjust and modify actions based on ecosystem responses are critical capabilities for local communities. These capabilities enable understanding and reading of the ecological feedback and rely therefore, on ecologically knowledgeable communities (Sundkvist et al. 2005; Reyes-García 2015; Loring 2021). Enhancing local peoples’ capacities through co-learning, sharing (e.g. Sitio Vale das Cabras), and building a collective understanding of their food system, along with its ecological and social context (e.g. Hudson River flows), is an integral part of this approach. Effective collective learning has been central to humanity's survival and is crucial to the long-term vitality of regenerative, inclusive food systems. One of the most important steps in any transformation process is the change of mindset and narrative (Mang et al. 2016; Anderson and Rivera-Ferre 2021), as these are the filters through which the world is perceived and experienced. They also influence which practices are adopted and applied (Mang and Haggard 2016).

Therefore, the fundamental premise behind this principle is that we, as humans, are part of a living system and through increasing our awareness and scientific understanding of living systems and being open to experiential learning by doing, we have the opportunity to change the current mindsets and extractive narratives currently associated with industrial food systems. Thus, helping us to learn how to enable RIFS to emerge and prosper.

## Discussion

### Advancing the discussion and action on FS regeneration

There is a radical and unprecedented need to disrupt, reconfigure, redesign, and regenerate our extractive food systems (Dahlberg 1993, 1994; Anderson and Rivera-Ferre 2021, Loring 2021). In this perspective, to advance the discussion on regenerating our food systems we first reimagined our food system, shifting the narrative from extractive to regenerative and inclusive. Furthermore, we developed five guiding principles that work within an interactive framework to support the transition and transformative processes required to create RIFS. Breaking imaginary “lock-ins” are fundamental in creating future alternative realities that we want to explore or achieve (Iles and Montenegro de Wit 2015; Marquardt and Nasiritousi 2022; Schmelzer and Büttner 2024). We have done this, also creating greater coherence in the vision through the use of the four nested outcome areas, scientifically underpinning them by building off available frameworks and literature (Supplementary, S1). These four outcome areas of RIFS will look very different depending on the place-based context they are being applied to. However, they should also not be seen as end points or a final destination, as they will require continuous revision and adaptation, based on the insights and learnings of a project or a community (Principle 5) for which agency and collaboration is required. While many visions can initially be seen as “utopian”, they still provide the opportunities for further creative and critical discussions, deliberation and dialogue. This is also what needs to be generated if we, collectively as members of society, are going to create regenerative imaginaries for our future FS. Our proposed vision of a future regenerative, inclusive food system RIFS has been presented here, because for a FS to function properly and to work for all, advancement in these four outcomes areas is what we argue is needed to create regenerative results.

The five principles support the transformative actions to enable the emergence of RIFS. They have been outlined and theoretically underpinned in the previous sections, building off the learnings and insights of other regenerative and FS initiatives. The number of principles comes from distilling the universal characteristics found in the other initiatives to five critical principles. It is imperative to keep the number of principles to a pragmatic and manageable number, to help those working with them to remember them relatively easily and to use them. These principles are not proposed as a rigid dogma, but rather as points for orientation to guide a reflective and adaptive approach in practice, that encourages discussion and experiential learning. They are to help shape and adapt the visions of RIFS, but they do not specify it. Examples of how these principles manifest themselves in practice have also been provided in this paper. These examples provide insights into the diversity of options and pathways available for creating RIFS. However, we acknowledge that to determine the true applicability and value of these principles will require participatory testing and co-evolution of the principles with communities (human and non-human) that suffer currently from degenerative and non-inclusive FSs. Integrating such perspectives will improve their applicability. Thus, it is also good to note, that while these principles have been developed across three East African contexts, they can be adapted and contextualised to any place.

Ultimately, the principles presented in this paper are about creating balance, empowerment and agency. To create the enabling conditions that return the regenerative capacities of our FS, building-in resilience through ensuring the benefits are shared equitably. Empowering all actors involved, from producers to consumers, to realise that they have agency in decisions been made and in their vision of a future FS (Coninx et al. 2022). In particular the principles are about creating opportunities to remove the barriers of people in vulnerable situations that lead to food and nutritional insecurity, as well as preventing people from being active in the FS (IFPRI 2020). Furthermore, it is also about giving nature a voice and a seat at the table when making decisions about its future, that nature itself is a stakeholder (e.g. understanding catchment needs of a river, so that it can “live” and thus provide life to those dependent on it) (Borràs 2016; Putzer et al. 2022; Putzer et al. 2025). We argue that when these principles are all occurring in a transition or transformation process, this provides the fertile ground for RIFS (see Table 2).

While we present the idealised descriptions of the principles (i.e. what they should look like or entail) implementing them will not be without challenges. Below we outline some examples of open questions which help to illustrate these challenges:

Principle1: How can locals be encouraged to connect more to nature, to the places where they live and to find a collective sense of purpose to create more social and nature care? Not everyone can relate to the places where they are living, either because they have immigrated to that place, or they have never really valued it. International companies sourcing raw materials from a place, while being a large driver of the activities shaping a place, may have no connection to a place or the people there, and see it only as one of many locations where they source their raw materials, how can they be convinced to invest their time and efforts in co-developing a sense of place and purpose with the locals?

Principle 2: How to encourage or facilitate large companies to reconsider their production practices to be more socio-ecological in design? This takes time, effort, and resources. How to generate the knowledge and support to help farmers adopt more socio-ecological practices? What actions need to be carried out to ensure that the burden sharing of farmers in such an undertaking is shared fairly? What business models exist to create this? How to encourage consumers to value such initiatives and the risks been taken? How can the scientific community and research institutes support the needs of food system actors trying to create positive change?

Principle 3: How to get the communities, farmers and companies to see the bigger picture of their actions (e.g. managing uphill forests contributes to protecting downstream flood plains from excessive or dangerous flooding). Should this be done through intrinsic motivation or through government instruments or compensation? How can trust be created between actors that may have historically been in constant disputes, that have no social capital with one another? How to ensure the optimum and healthy configurations of networks (connections) to ensure resilience, communication flow and support?

Principle 4: How to introduce and encourage elements of land and nature stewardship as part of polycentric decision-making in heavily bureaucratic states or countries? How to deal with power disparities and decision-making when it comes to the places where a project is being implemented? How to convince governments and businesses to prioritise the long-term benefits of RIFS over short term profits? How to deal with gender inequalities within the household in order to ensure a fair allocation of benefits and a fair distribution of labour?

Principle 5: How to create a process where room for learning and adapting is encouraged?, How to encourage the communities involved to track and monitor their own change and be custodians of their own transition and learning? How to advocate for funding structures to allow the scientific community to learn more from failures and to allow for work that is still valued even if it is not peer-reviewed?

There are many open questions on the realities of implementing actions to encourage these principles to emerge in a transformative process, particularly in relation to time, money and effort. Additionally, it is difficult to currently ascertain if these principles really do, as we propose, stimulate progress towards RIFS, the empirical evidence still needs to be collected. While some answers are becoming clearer (see section below: Putting theory into practice (it’s a living process)), many require time to come into fruition. However, further discussion on this is outside the scope of this perspective paper. Regardless, it is important to ask these questions if we want to bring the principles we are proposing to life. Additionally, while there are still many obstacles and challenges, Table 2 provides the evidence that “small wins” (Termeer and Dewulf 2018) in this direction are happening and can be learnt from and grown further.

In this paper we have so far outlined the vision for RIFS, and the associated narrative structured with the four RIFS outcomes (Fig. 1). However, the RIFS principles work within an interactive framework to support the transition and transformative processes and strategies required to create RIFS. This we explain in the succeeding sections.

### RIFS principles used as part of a dynamic framework

**Fig. 3:**
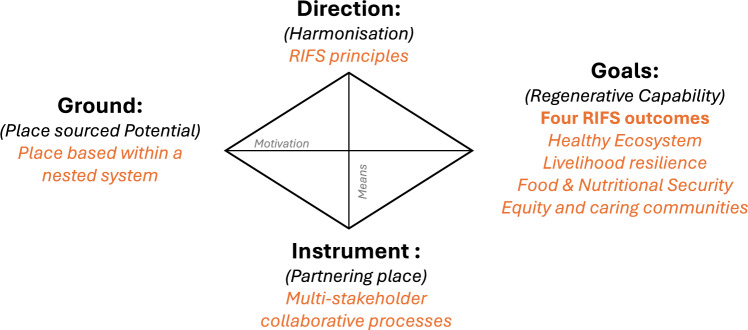
RIFS framework with the four interacting components of change, adapting the framework of Mang and Reed (2020), with permission

Through reading the available literature (see sections above, Table S3.1) on regenerative development we returned time and again to the philosophies, teachings and frameworks of the Regenesis Institute (Mang and Haggard 2016; Mang and Reed 2020). From this it was identified that the Tetrad framework outlined by Mang and Reed (2020) could be used to mobilise RIFS in a place-based approach (Fig. 3). The framework provides four key elements that are crucial for regenerative development projects. It works as a system to integrate and align the motivation and means for regenerative outcomes, but it is also designed as a framework to allow methodologies and approaches from other scientific disciplines to be integrated and incorporated into the design of the regenerative FS project. Going from left to right, the two elements define the motivation (e.g. energy and will) and the desirable future state (goal) of the project. The key elements from top (principles) to bottom (means/instrument) provide guidance and structure on how the project is carried out, ensuring that from start (ground) to end (goal) a balance is kept between all elements to ensure the process works. Among other things, this means maintaining motivation to achieve a regenerative result, as well as a mindset and capacity to continue working on nurturing the “result”.

*Place and potential (ground)*: for regenerative projects, which are place-based approaches, a fundamental activity is to develop a holistic understanding of place. What makes a place unique and what role does a place and the communities living there play in the bigger picture (e.g. river catchment, forest ecosystem, region).

*Four RIFS outcomes (goals)*: this relates to the potential of place and the regenerative capacities of communities in place. The ultimate goal for RIFS is to build the regenerative capability of the living systems associated with the place (ground), using the four outcomes identified in Fig. 1 and explained in more detailed in section “The vision for RIFS, using four nested outcome areas”. However, it needs to be emphasised that how RIFS evolve in different places will be specific to a particular place and the communities (human and non-human) living there. While there will be universal similarities between locations, RIFS will be unique to each place and community.

*Partnering place (Instrument):* regenerative projects need to tap into the *motivation* or the will of a community^24^ to achieve RIFS. Partnering with local communities and stakeholders to build such regenerative capabilities can be done through multi-stakeholder collaborative processes (e.g. Living Labs), as these can create the spaces that enables and fosters co-creation processes for social innovations sourced from place. Such activities can create an energy around a common sense of purpose that can be used to empower and create change.

These four components all need to be brought into alignment, where each needs continuous attention in order to enable the emergence of regenerative, inclusive food systems (Mang and Reed 2020). The principles, as introduced in this paper, are especially important as they act as a compass to navigate the complexity of place-based and regenerative work.

### Working towards transformative change

While we outline principles here to be used within a dynamic framework to help guide this transformation, there are many wicked challenges and barriers involved in the food system that need to be overcome to lead to the deep transformative change that is needed (Loorbach 2022). Developing regenerative pathways towards achieving the goals of RIFS depends first on a change of mindset and world view, a form of transformative learning (Burns 2015).

**Fig. 4:**
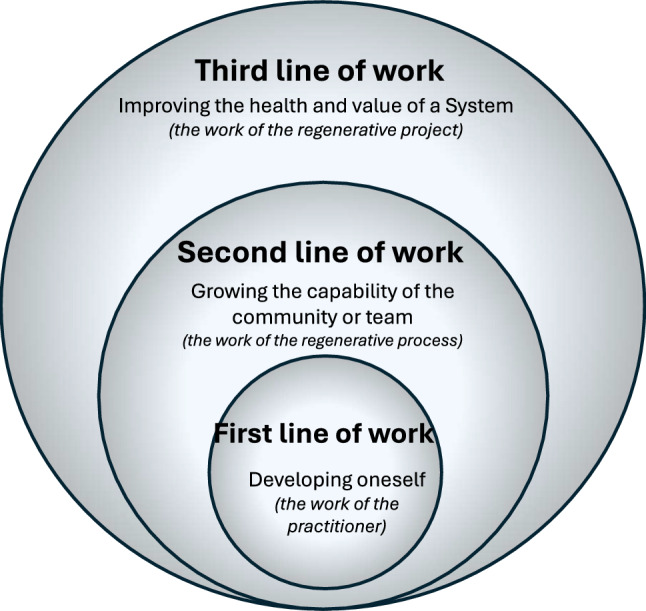
Graphical representation of the three lines of work (adapted), used with permission from the Regenesis Institute

Indeed, regenerative change will be a challenging process, as it essentially starts within ourselves and our mindsets. Shifting mindsets and changing behaviour is notoriously difficult but there are ways to support it. Therefore, for these principles to be truly effective in creating transformational change they need to be implemented using the three lines of work (Fig. 4) as proposed by the Regenesis Institute (Mang et al. 2016). Thus, as a first step this means that they can be used to support the needed mindset change (first line of work), through the exploration and experiential learning of trying to apply the five RIFS principles in any process or project undertaken (second line), by anyone engaged with and working within the food system to ultimately lead to regenerative and long-lasting improvements in the food system (third line). This can include, for instance, researchers, farmers, community members, employees and CEOs of international companies (see Tables S4.1–S4.5). One generic example of this experiential learning (second line of work, Fig. 4) could be in a multi-stakeholder participatory setting where people start connecting to one another and to each other’s ideas (Principle 3), lift these ideas up to a new sense of life and potential, and then link them to practices that express their care for community and nature (Principle 1 and 2). In the process everyone is included in the discussion, and everyone is given space and a voice to raise both their concerns and their aspirations (Principle 4). From these exchanges and interactions, they learn how to work with each other and why working with one another can be beneficial for themselves, their community and for nature (Principle 1 and 5). While this example is described as a linear progression, the principles represent an integrated whole. Once the five principles are internalised through repetition and training they will show up organically and iteratively as a natural expression of relatedness.

Another important element for creating this change will be through the engagement of the scientific community. This will be particularly relevant in relation to bringing together the many different types of knowledges^25^ required for transformational change in a legitimate manner that provides credibility to different forms of relevant and salient knowledge (Cash et al. 2003).

Each scientist and scientific institution need to reflect on their role, position, actions, and influence in order to engage more meaningfully in a fair and just FS transition. It may lead to some tensions, as it may require changing the operational norms within which the research community work, requiring levels of institutional transformation (Kok et al. 2019). This will require us to ask particularly pertinent, if difficult questions. It requires us, the scientific community, to start engaging in the FS using the RIFS principles combined with the three lines of work. This provides opportunities to also transform the way research is conducted (see supplementary material S4). At the first and second lines of work pertinent questions could be, but not limited to: what is my purpose for being involved in a project? Does my role in this project contribute regeneratively by giving back, giving energy to a place, to a community, to my team, to me? Am I thinking of developing and designing innovations founded on socio- ecological design (e.g. what are the community needs and is what they are doing matching to place, are they circular?). Am I building connections between the necessary people to encourage un-siloed and unified thinking? Within my projects or my team, am I ensuring that everyone has an equal voice and is heard? Do I allow space for creating joint understanding between different disciplines, cultures? Am I open to learning by doing, co-working and developing concrete approaches, innovations with a diverse team? At a broader systemic level (third line of work), such questions could be: how are scientific systems working globally? How are they organised? Who is benefiting from the knowledge generated? Are both scientific and traditional agroecological knowledge treated the same and, if not, why not? Furthermore, are there disparities between the benefits of knowledge and research money going to the global north, while many of the issues are occurring in the global south? How do we equalise our roles for tackling these knowledge gaps together? (Tschersich and Kok 2022). Additionally, how can we facilitate processes to build evidence and examples of the general efficacy of regenerative practices? Do we need new types of processes to encourage trust? Is there space, trust and time provided by project funders for experiential learning and new forms of conducting science-based interventions?

The three lines of work has the potential to allow the RIFS principles to be adapted to different contexts which aim to have a regenerative result (see supplementary material S4). This is what makes these principles unique, as the focus is not just on directing the change of a particular project, it also focuses on the change within individual actors themselves, guiding them and their actions in that system to create regenerative results.

### Putting theory into practice (it’s a living process)

The REFOOTURE project (Adokorach et al. 2023c), which started in 2020, is a collaborative project between Muni University in Uganda, Egerton University in Kenya, Wageningen University & Research in the Netherlands, and Stichting Wageningen Research Ethiopia (SWR Ethiopia) in Ethiopia, with contributions from Jimma University and Bahir Dar University in Ethiopia. The project aims to contribute to food systems transformation by fostering Regenerative, Inclusive Food Systems (RIFS) in three East African countries: Ethiopia, Kenya and Uganda. The RIFS principles have been developed in the frame of this action-research project. Due to the differing scientific disciplines, expertise and nationalities members had very divergent views and understanding of what RIFS should be like or consist of. The co-development of a RIFS vision, principles and dynamic framework created a clarity that allowed us to work more structured and systematically in very complex and dynamic transition processes (Supplementary material, S5). This was a fundamental requirement because REFOOTURE emphasises the collaboration of three critical forces that all play a role and have a stake in the regenerative process. These are: *the community* (the people, including the marginalised and disadvantaged within that community), *the powerholders* (e.g. government entities and policymakers, co-operatives and local businesses), and *nature* itself (e.g. the place). By bringing these critical forces together, the project aims to create synergies that foster “collaboration for regeneration” of a community, its place and the associated FS (Reemer et al. 2023). Thus, creating a win–win-win approach, where all three forces share the benefits and burdens, and risks of the transformation are distributed fairly. The principles and dynamic framework enable the development and design of the collaborate to regenerate participatory processes and engagement (C2R) to create a win–win-win for all three critical forces. Furthermore, they are enabling the structured development of a toolbox for establishing and collecting (non-extractive) evidence of how regenerative practices and principles can lead to the four RIFS outcomes in the places and FS where the project is active. While the authors of this paper actively use the principles in the project (ongoing at the time of writing this paper), we will use the REFOOTURE project to further reflect on the applicability and value of the principles.

The REFOOTURE project is a placed-based project; however, the places within which we are working are nested within bigger regions and within their countries. This is why we are also striving to build connections with like-minded stakeholders at different levels, while keeping the unique approach of RIFS and its regenerative potential. These collaborations and experiential learning from using the principles in the FS transformation will bear fruit in the long term and we envision them being a series of “small wins” that create the bigger momentum for change (the win–win-win). Right now, if we manage to prepare the soil, plant the seeds for change and create the regenerative capacity for growth, then imaginaries might just become realities in time.

## Conclusions

The (ideal) outcomes of a FS is to ensure food and nutritional security for all people, while supporting decent livelihoods, creating caring communities, all thriving in healthy ecosystems, through which, resilience to shocks and stresses can be established. However, our current FSs are failing us, and these desirable outcomes are not being achieved. Therefore, there is a pressing need to reimagine, reconfigure and regenerate our FS. The aim of this perspective paper was to further the discussion on regenerating our food system, by describing our future FS vision to be regenerative, inclusive food systems and providing five guiding principles that work within an interactive dynamic framework to achieve the four RIFS outcomes. The principles can help to reconcile emerging tensions among the four defining outcomes of RIFS. We argue that when all five principles occur in a transformation process, this provides the fertile ground for RIFS. The principles are nested, meaning in any RIFS process all five principles need to be experienced either simultaneously or in different iterations of succession, so as to reveal actions and opportunities for transformational learning that will genuinely contribute to the regenerative capacities of these FSs. This perspective paper provides the theoretical underpinning for the RIFS vision and principles and the approach for regenerative development which has been taken by the REFOOTURE project but is not restricted to it. It is a placed-based project, ongoing at the time of writing this paper, which aims to contribute to food systems transformation by fostering Regenerative, Inclusive Food Systems (RIFS) in three East African countries: Ethiopia, Kenya and Uganda. While REFOOTURE focuses on places in East Africa, the RIFS vision and principles can be adapted and contextualised to any place. Taking Europe as an example, the insights of this paper on RIFS principles can be useful to support and contribute to political, scientific and societal discourses which are emerging on regenerative development,^26^ regenerative agriculture^27^ and agroecology.^28^ The unique aspect of these principles is that they are not to only direct activities or direct change related to a particular project, but rather they also focus on the actions of individual actors themselves, guiding them and their actions in whatever systems they are working to create regenerative results. Therefore, we encourage and invite others to also test and experience the RIFS principles, as the potential value and evolution of these principles to create positive changes in our FS will be through the associated testing and experiential learning.

## Supplementary Information

Below is the link to the electronic supplementary material.

Supplementary file1 (PDF 1288 KB)

## Data Availability

All data supporting the work outlined in this study is freely available and can be found in the reference list provided at the end of this article and in the supplementary materials.

## Footnotes

1 Definition of socio-ecological systems according to Folke et al. (2016) “the social refers to the human dimension in its diverse facets, including the economic, political, technological, and cultural, and the ecological to the thin layer of planet Earth where there is life, the biosphere. The biosphere is the global ecological system integrating all living beings and their relationships, humans and human actions included, as well as their dynamic interplay with the atmosphere, water cycle, biogeochemical cycles, and the dynamics of the Earth system as a whole”.

2 “The Five Capitals Model provides a basis for understanding sustainability in terms of the economic concept of wealth creation or ‘capital’. Any organisation will use five types of capital to deliver its products or services. A sustainable organisation will maintain and where possible enhance these stocks of capital assets, rather than deplete or degrade them.” (https://www.forumforthefuture.org/the-five-capitals).

3 Our foodsystem, “our” here refers to all of society and the respective food system(s) where we live, from those that produce food to those that consume it.

4 According to Reyes-Garcia (2015), “Traditional ecological knowledge (hereafter TEK) is a dynamic knowledge system that emerges from people’s interactions with their environment and that is embedded in local institutions…(and) to remain effective, it requires well connected social networks and supporting institutional frameworks and that communities have integrated formal and informal mechanisms for flexible and rapid decision-making that evolve through institutional learning organizations”.

5 “Feedback loops can act as control devices in both natural and SES. Negative loops counterbalance change and have a stabilizing effect, such as the regulation of our body temperature, while positive feedback reinforces change and amplifies rather than reverses change” (Sundkvist, et al. 2005).

6 Community here also refers to the human and non-human parts of ecosystems supporting the FS.

7 A sustainable FS according to the FAO is: “A FS that delivers food security and nutrition for all in such a way that the economic, social and environmental bases to generate food security and nutrition for future generations are not compromised. This means that: it is profitable throughout (economic sustainability); It has broad-based benefits for society (social sustainability); and it has a positive or neutral impact on the natural environment (environmental sustainability)” (FAO 2018a).

8 According to Marquardt and Nasiritousi (2022), “Imagination is a fundamental and empowering element for people and society, as it has the power to enable the creation of new forms of life. Imaginaries are then the collective imagination or visions shared more widely within society. These imaginaries are shared or contested visions of the future, and they shape narratives, policies, institutions as well as construct collective identities. Essentially they are a key building block for creating our social order “. We use vision and imaginary interchangeably in this paper.

9 Assumed here to be similar or the same as socio-ecological.

10 “We define inclusion as a focus on the weakest and most vulnerable, as it relates to access to and affordability of food, and employment and wages in the FS” Gaupp et al. 2021.

11 We refer here to the five capitals (see footnote 2).

12 Value-adding can be defined here as changes to the FS that improve the state and capacities of such systems in a way to reach the interconnected system goals.

13 According to Weick et al. (2005), “Sensemaking involves turning circumstances into a situation that is comprehended explicitly in words and that serves as a springboard into action”. It is both a retrospective and prospective process to: help make sense of complex situations; to support the recognition of patterns that can be used to help inform decisions; to support categorising experiences and understandings for them to make sense and to take effective action. It is about working with ambiguity to begin with to create something more concrete. It is systemic and about organising through communication.

14 Documents on: Lessons from the first, second and third round table dialogues on living labs in east Africa available online: https://www.wur.nl/en/project/refooture-food-futures-eastern-africa.htm.

15 All actors mean here, all those relevant and involved in their respective food system, from producers to consumers.

16 Here we use the definition of social innovation of Baselice et al. (2021), it is “the reconfiguring of social practices, in response to societal challenges, which seeks to enhance outcomes on societal well-being and necessarily includes the engagement of civil society actors. This definition emphasises the key role of human agency in transforming existing attitudes, social networks, or governance arrangements to ameliorate the collective well-being of the community”.

17 Here we use the definition of Secco et al. (2020),..“attitudes are a predisposition or a tendency to respond positively or negatively towards a certain idea, object, person, or situation. They are linked with culture, social norms, opinions, beliefs, ethical and moral values (individual or shared), and discourses. People’s attitudes are affected by the socio-economic context and historical development trajectories in which they live, as well as by states of feeling, emotions and are contingent to personal circumstances. Attitudes influence the collective and individual choices of action, and responses to challenges, incentives, and rewards”.

18 Coninx et al. (2022) have also developed an operational framework to support dialogues between actors aiming to initiate a transition in a FS, with the aim of guiding them on how to jointly include and operationalise aspects of the four justice concepts, distributive, procedural, recognition and restorative seen to support a fair and just FS transition. For further information on the transdisciplinary project underpinning this framework please see website here: https://research.wur.nl/en/projects/1-1d-5-just-transition-kb-34-004-017.

19 https://ecojurisprudence.org/initiatives/.

20 Governance here refers to all processes of governing in relation to social practices and activities, such as those, for example, in families, tribes, networks (formal and informal), as well as governments (Bevir 2012). Additionally, it must be noted that “Governance is inherently political: as a result of conflicting interests and power imbalances, FSs fail to deliver equitable and just access to food. Moreover, the impacts of shocks and stressors are not evenly distributed across actors in the FS. There are significant differences in vulnerability and response capacities between different groups of people, sectors and regions” (de Steenhuijsen Piters et al. 2021).

21 “People may be perpetually locked into resilient but undesirable states of poverty and marginality” (Tanner et al. 2015) or the socio-technical regimes of industrial FSs can have internally stabilising features that keep these regimes functioning (e.g. subsidies, concentration of power), so these systems are per se resilient but are not always entirely beneficial (Loring 2021).

22 Livelihood assets are identified as the 5 capitals (natural, social, human, financial and physical capital).

23 According to Mehmood et al. (2020) “transformative learning as an approach to experiential pedagogy with focus on education and learning based in local communities and the surrounding places; second, experiencing place through sense-making to help people relate closely to their values and meanings of place; third, regenerative action to reverse and recuperate from the negative impact of humans on the environment and promote place stewardship. Through a dynamic combination of these processes, new socially innovative agency is created”.

24 Community here refers the collective of human and non-humans in a place.

25 Knowledge can exist in many valid forms, such as local, indigenous, and traditional, and can exist and be combined with positivist knowledge forms (e.g. knowledge that is juridical and scientific in character) (Iles and Montenegro de Wit 2015).

26 https://www.health.belgium.be/en/regenerative-development-high-level-event-report.

27 https://eara.farm/.

28 https://www.agroecologypartnership.eu/.
