# Supplementary material for: Regenerating the food system: A proposed vision and guiding principles for regenerative, inclusive food systems (RIFS)
Source: Ambio. 2025 Dec 29;55(7):1493–519. doi: 10.1007/s13280-025-02319-1 (PMC13230463; doi:10.1007/s13280-025-02319-1)
Supplement: Supplementary file 1 — Supplementary file1 (PDF 1288 KB) [file 13280_2025_2319_MOESM1_ESM.pdf]

Ambio

Supplementary Information

This supplementary information has not been peer reviewed

Title: **Regenerating the food system: A proposed vision and guiding principles for Regenerative, Inclusive Food Systems (RIFS)**

S1. Four outcomes areas for RIFS

Here we provide a brief description of important aspects related to the four outcome areas. For each aspect descriptions and some characteristics to explore are provided. The latter is not a complete list, but to provide "rough" guidelines for practitioners in the field to make observations during different engagements with the communities they are working with (i.e., like a rapid assessment tool). These aspects have been piloted in the field as part of the REFOOTURE project. Please refer to Reemer et al., (2023) for further insights.

Table S1.1. Food and nutritional security dimension derived from HLPE, 2020

Aspects	Descriptions[1]	Characteristics to explore
**Availability**	*Having a quantity and quality of food sufficient to satisfy the dietary needs of individuals, free from adverse substances and acceptable within a given culture, supplied through domestic production or imports.*	• People are producing themselves; food is produced sourced locally, larger FS networks, good trading conditions in region?<br>• People are eating a diversity of food for a balanced diet (protein, fat, carbohydrates)?<br>• People can purchase culturally relevant food; can it be sourced/grown locally?<br>• What is happening with food waste or is there a tendency for high food losses (why)?
**Access**	*Having personal or household financial means to acquire food for an adequate diet at a level to ensure that satisfaction of other basic needs are not threatened or compromised; and that adequate food is accessible to everyone, including vulnerable individuals and groups.*	• People physically gain access to food- that there is food available (e.g., they can grow or get food) – proximity to markets or produce?<br>• People able to afford food- they can buy the necessary amounts of food to feed everyone in the family, especially women and children.<br>• Social access to food – even vulnerable people can acquire appropriate levels of food?<br>• Women and children have access within the household?
**Utilisation**	*Having an adequate diet, clean water, sanitation and health care to reach a state of nutritional well-being where all physiological needs are met.*	• People have the means to cook (e.g., energy, facilities)?<br>• People have access to fresh water to prepare, cook food?<br>• People can store food properly and safely.<br>• People prepare food safely?
**Stability**	*Having the ability to ensure food security in the event of sudden shocks (e.g., an economic, health, conflict or climatic crisis) or cyclical events (e.g., seasonal food insecurity).*	• Are there issues relating to climate change (e.g. crop losses, soil loss, unusual pests)?<br>• Is there a lack of supply due to other reasons (e.g., conflict, economic crises)?<br>• Is their income too variable, i.e. loss in earnings for a time?<br>• Can they keep food in storage/reserves (e.g. in their own garden/holding)?
**Agency**	*Individuals or groups having the capacity to act independently to make choices about what they eat, the foods they produce, how that food is produced, processed, and distributed, and to engage in policy processes that shape food systems.*	• Are there factors limiting choice preference for food?<br>• Gender inequalities in the household?<br>• Lack of knowledge – that people have their own agency (in relation to food)?<br>• Bias in government policy, trade deals that affect people on the ground?
**Regenerative practices**	People are conscious of where their food comes from, they are conscious that their activities can enhance nature and this is what they try to do, to diversify rather than intensify or to be extractive instead– they also give back.	• Regenerative food production (agro-ecological practices, conscious of water and soil health)?<br>• Use of organic fertilisers and pesticides?<br>• Awareness about safe food processing, storage and transport?<br>• Water harvesting – in a non extractive manner?

1. Definitions derived from HLPE, 2020

Table S1.2. Livelihood resilience dimension: Buffer capacity[1] (Adapted from: Ifejika Speranza, Wiesmann et al. 2014 and Jacobi, Mukhovi et al. 2018)		
**Aspects**	**Definitions[2]**	**Characteristics to explore**
**Diversity** *(of crops and breeds, diversity in operations and activities)*	The diversity of crops and breeds on farms, and/or in markets, and/or consumed.... Resilient systems are diverse systems, and diversity means that a loss of one resource may be compensated by another.	• Are there more than 3 crops, different varieties adapted to local conditions?<br>• Multi-, poly- or inter-cropping, permaculture?<br>• Different animal breeds adapted to local and changing climate conditions?<br>• Farming considering other non-productive species (co-growing with nature e.g. using native species with deep tap roots for water harvesting)?<br>• Have different revenue streams from a diversity of activities?
**Natural capital**	Natural capital can be defined as the world's stocks of natural assets which include geology, soil, air, water and all living things. Key characteristics: Water, soil, ecosystems, forests, pastures, energy, geology and land.	• Who owns the Land, what are the land rights/ownership?<br>• Access to irrigation water, is it a sensitive water source?<br>• Relationship to natural resources being used are respectful and responsible.<br>• Animals being farmed with animal welfare considerations, is it respectful?
**Human capital [3]**	The knowledge, information, experience, ideas, skills, and health of individuals. Recently, the definition has assumed a more collective oriented meaning (i.e., the collective skills, knowledge, or other intangible assets of individuals that can be used to create economic value for the individuals, their employers, or their community).	• What level of literacy does the community have?<br>• What level of indigenous knowledge, practices do they have?<br>• What is the level of skills and capabilities are the women displaying in the group?<br>• What are the skill and knowledge levels of the group regardless of literacy levels etc.?<br>• Are people showing innovative techniques, business acumen?
**Financial capital**	Any economic resource measured in terms of money used by entrepreneurs and businesses to buy what they need to make their products or to provide their services to the sector of the economy upon which their operation is based, i.e., retail, corporate, investment banking, etc.	• Are all (household) income sources related to the food system or are there other revenue streams?<br>• Are people earning enough to cover their costs?<br>• Can they save money – is this a possibility, if not why?<br>• Is it possible for them to get a loan or other financial support e.g. through community "table banking"?<br>• Do women have financial stability or are there issues?
**Social capital** *(this describes the configuration of the connections)*	Networks together with shared norms, values and understanding that facilitates cooperation within or amongst groups. May have "bonding" or "bridging" functions.	• Do people have a good relationship with their family?<br>• Do people have a good relationship outside their immediate family members? (e.g. neighbours, greater community)<br>• Are people organised in clubs, common interest groups?
**Physical capital**	This can also be seen as human constructed resources (built) or Infrastructural Capital. This refers to the basic physical and organisational structures and facilities (e.g., buildings, roads, power supplies) needed for the operation of a society, enterprise or area. (important for LNOB, table S1.6)	• What is the condition of housing and other installations?<br>• Do people have access to inputs, machinery and tools necessary for the system to function?<br>• Does the place have access to transport, transport networks?<br>• Are there sanitary services: water, electricity, gas, waste manage?<br>• Schools and health facilities, what state are they in?
**Cultural Capital**	"Shared attitudes and mores, which shape the way we view the world and what we value. Some aspects are closely related to human and social capitals".	• Are the people sharing stories, songs etc.?<br>• Are cultural events organised in the area?<br>• Are there different minority local languages, clans, groups?<br>• Can customs /traditions be clearly recognised?
1. Buffer capacity refers to: Buffering capacity here refers to the capacity of the food system to absorb disturbances (e.g., climate change, policy changes) and cushion change that in a way that can use (Ifejika Speranza, Wiesmann et al. 2014, Jacobi, Mukhovi et al. 2018, de Steenhuijsen Piters, Termeer et al. 2021) "*the emerging opportunities to achieve better livelihood outcomes such as reduced poverty*"(Ifejika Speranza, Wiesmann et al. 2014)<br>2. Most definitions and characteristics for the various aspects are taken from (Secco 2020), diversity is taken from the Tool for Agroecological Performance Evaluation, TAPE (FAO, 2019)<br>3. Here we look at in the perspective of local scale delivery of resources – extractive processes, below we look more at the systemic level)		

**Table S1.3. Livelihood resilience dimension: Self- Organisation[1] (Adapted from: Ifejika Speranza, Wiesmann et al. 2014 and Jacobi, Mukhovi et al. 2018)**		
**Aspects**	**Definitions [2]**	**Characteristics to explore**
**Institutions Role**	Enforcement of rules and regulations governing land and water use (e.g., applied sanctions for non-compliance); institutions encourage collective action (e.g., support /partnerships with farmer, food system organisations)	• How are existing rules and regulations governing land and water use being enforced (if at all)?<br>• What are peoples' relationships with various institutions?<br>• Is there a sense of skewed power dynamics? Is there a sense of trust/distrust of the institutions?
**Cooperation, Reciprocity and trust** *(this describes the relationship of the connections)*	Refer to interactions between actors in the SES resulting in the creation of own rules, norms and values (institutions), building trust and decreasing dependence on external actors for information, innovation and capital	• People recognise themselves as part of a group, are in a group with the other members present?<br>• People talking openly – is there a general sense of trust, is there laughter?<br>• Are there a number and type of associations, groups, communities where people are members (networks)?<br>• Can the characteristics of the networks people be part of be clearly linked to place?
**Ability to be "Circular".** *(reliance on own resources)*	One of the core premises of ecological design is the need to transition our social systems to a greater reliance on renewable resources through recycling, reusing and reducing (No fossil inputs). We need to be able to mimic the fundamental designs, networks and structures of nature, while at the same time enhance those diverse natural systems in which we will become more dependent on and becoming more aware of ecological feedback	• Reliance on local resources, short supply chains?<br>• Reduced dependency on input and commodity markets?<br>• includes indigenous knowledge, elements of biomimicry and more nature-based solutions?

1. Self -organisation refers to how food system actors are capable of having certain levels of autonomy , are free to act (agency), are free to instigate collective action, are independent and decentralised, are self-reliant and are capable of controlling certain food system process and self-regulate themselves in order to ensure the food system is functioning properly. Thus, helping to foster trust and contribute to the empowerment of food system actors (Ifejika Speranza, Wiesmann et al. 2014, Jacobi, Mukhovi et al. 2018). "Self-organisation highlights how human agency, adaptive capacities, power and social interactions shape social resilience". (Ifejika Speranza, Wiesmann et al. 2014),
2. Definitions come from: (Ifejika Speranza, Wiesmann et al. 2014), "Except ability to be circular" this is derived from the follow sources: 1(Shu-Yang, Freedman et al. 2004, Sundkvist, Milestad et al. 2005, Mang, Haggard et al. 2016),

Table S1.4. Livelihood resilience dimension: Capacity to learn and be adaptive[1] – (Adapted from: Ifejika Speranza, Wiesmann et al. 2014 and Jacobi, Mukhovi et al. 2018)		
**Aspects**	**Definitions**	**Characteristics to explore**
**Knowledge of threats and opportunities**	Ability to perceive and be aware of and analyse, risks, threats/potential opportunities	• People show examples of awareness of potential risks and perceive these risks to be important for them?<br>• Scale at which the risks are perceived (day-to-day risks, local, somewhere else)?<br>• Are the perceived risks/threats a real risk/threat?<br>• Can see opportunities from potential changes in their situation?
**Reflective and shared learning**	Access to and quality of learning activities; Food system actors proactively create desirable futures based on experience rather than simply react to present conditions	• Participation in courses such as farmer field schools?<br>• Participation in community development activities (e.g., caring for local area, helping neighbours) and using these skills/learning to help further?<br>• Commitment to learning?<br>• Actively reflect on what they have learnt.<br>• Do they adapt their actions based on what they have learnt?
**Functioning knowledge networks**	Relevant knowledge is being passed along to the right people, knowledge is shared and transparent, the knowledge being shared is helping to create opportunities, build trust and confidence between the various members of the network and it is creating a solid foundation for mutualistic relationships	• Are people sharing information about meetings and opportunities with each other?<br>• How are people connecting, physically (in person) or digitally -are they connected through modes of telecommunications or social media?<br>• Is information being passed on to people not present at meetings?<br>• Is beneficial information being shared between people, or is information "being kept" by certain people (i.e. not passed on)?
**Existence and use of local traditional knowledge**	Biological and cultural memory, identity and knowledge embodied in a system and its components. An important factor used here as a proxy is: (1) existence and (2) use of local traditional knowledge.	• Recipes for local dishes?<br>• Songs, stories handed down.<br>• Maintenance of heirloom species?<br>• Combining traditional forms of cultivation with adaptations with new technologies that are beneficial?<br>• Combining new knowledge sources with more local traditional ones?<br>• Consultation and involvement of elders, older generation?
**Shared vision**	All actors /participants have a shared vision in the potential they see for the place they live, for their livelihoods and for the role they can play in achieving their join vision	• Are there differences between social groups in their ability to have and communicate individual aspirations?<br>• Is there discrimination visible e.g. women not included?<br>• Does the vision create a "common" sense of purpose?
1. Capacity for learning refers to an adaptive style of management, where previous experiences are reflected upon in context and incorporated into current actions and planning. The capacity to act, can be at the individual level, to multi-actor level (e.g., user groups) or at the system level (Ifejika Speranza, Wiesmann et al. 2014, Jacobi, Mukhovi et al. 2018, de Steenhuijsen Piters, Termeer et al. 2021). Capacity for learning and adaptation is crucial for long term food system resilience and livelihood resilience, as it enables people to also be able to anticipate and buffer against certain shocks. To date many of the discussions on food system resilience have been at system level, with limited attention been given to understanding better the role of the human capacity for learning and adaption "which is at the heart of a resilient food system" (de Steenhuijsen Piters, Termeer et al. 2021)<br>2. Definitions and characteristics have been derived from the studies of: Ifejika Speranza, Wiesmann et al. 2014 and Jacobi, Mukhovi et al. 2018.		

Table S1.5. Ecosystem Health Dimension[1]		
**Aspects**	**Definitions[2]**	**Characteristics to explore**
**Soils /paedology** (soil Health)	"The ability of the soil to sustain the productivity, diversity, and environmental services of terrestrial ecosystems" FAO- ITPS (2020). Schulte et al. (2014), refers to these soil services (ecosystem services) as soil functions, of which they identified five soil functions as being important for agricultural land use. These are: (1) Primary production; (2) Water purification and regulation;(3) Carbon cycling and storage;(4) Functional and intrinsic biodiversity, and (5) Nutrient cycling and provision	Are there:<br>• Visible signs of erosion (rills or gullies)?<br>• Visible signs of Puddling?<br>• Landscape design or activities to reduce erosion (e.g., terraces, cover crop, agroforestry)?<br>• Use of compost, manure, organic materials on soil?<br>• Soil colour (relative to what it should be)?<br>• Reports of soil erosion being a problem?<br>• Awareness of how to manage soils?
**Clean Water/ natural Hydrological systems[3]**	Clean and healthy water is essentially "*water that will not harm you if you come in contact with it*"[3.]<br><br>The hydrological system refers to a continuous exchange of water among the lithosphere, the atmosphere, and the biosphere (Narasimhan 2009). "*The concept of the hydrological cycle is quite simple. But its importance to life on earth is profound. The hydrological cycle plays an overarching role in the cycling of solar energy, sediments, and chemical elements vital for life.... life is simultaneously a product of the hydrological cycle and a factor causing changes in the cycle*" (Robertson, Perlman et al. 2022)	• Colour and transparency of water body is appropriate (e.g., it is not brown, there is no pearlescent sheen)?<br>• Smell in and around the water body is appropriate (e.g., it does not smell of stagnancy, sewage)?<br>• No visible sign of algae overgrowth in water body?<br>• reports of people being sick after drinking water?<br>• Issues raised about water during discourse?<br>• People aware of where their water comes from?.<br>• Aware of the hydrology of the place?
**Clean Air/atmosphere**	Air pollution is contamination of the indoor or outdoor environment by any chemical, physical or biological agent that modifies the natural characteristics of the atmosphere (WHO definition)[4]	• Is it difficult/uncomfortable to breath (e.g., air heavy with exhaust emissions from vehicles, fuel burning)?<br>• There are people burning fire or rubbish in the area.<br>• There is odour pollution (e.g., strong smells of animal wastes, faeces, rotting wastes, rotten egg smell)?<br>• People mention the smells.<br>• They have a knowledge of carbon emissions, also in relation to climate change?
**Biota and habitats** (Flora and Fauna of a given place)	The plants, animals' species and their communities that make up a particular habitat or ecosystem.	• The habitat looks degraded (e.g., patchiness in a forest, bald patches in a grassland, poor species diversity then expected)?<br>• Few species or evidence of species present (that should normally be present)?<br>• People talk about or have observed changes in wildlife?

1. A healthy ecosystem is one that has the ability to maintain its structure (organisation) and function (vigour), especially in relation to the ecosystem services it provides to humans and non-humans alike, over time regardless of external stresses (resilience) (Burkhard et al., 2008; and Costanza et al., 1999).
2. Definitions are derived from different sources and provided in text.
3. https://healingwaters.org/how-to-define-clean-water/
4. WHO definition reported here: https://www.afro.who.int/node/5526.. "Freshwater health" is defined here as "the ability of freshwater ecosystems to deliver ecosystem services and benefits, sustainably and equitably, through effective management and governance". (Vollmer et al. 2018)

Table S1.6. Equality and caring community Dimension		
**Aspects**	**Definitions [1]**	**Characteristics to explore[2]**
**Discrimination**	What biases, exclusion or mistreatment do people face based on one or more aspect of their identity (ascribed or assumed), including prominently gender as well as ethnicity, age, class, disability, sexual orientation, religion, nationality, indigenous, migratory status etc.?	• People with disabilities, young people or elderly being left out (e.g., left at the back of the group)?<br>• People in the group always being designated the tougher or less appealing jobs?<br>• People care for the elderly? Include them in the community?<br>• People being vocal against others?<br>• Bias in the group towards men, not recognising the labour contribution of women?
**Geography:**	Those who endure isolation, vulnerability, missing or inferior public services, transportation, internet or other infrastructure gaps due to their place of residence?	• There is no investment in infrastructure (e.g., poor roads, no schools, waste collections)?<br>• There is no Investment in education supplies (e.g., school materials)? Difficult for community children to get to the school?<br>• No medical centres nearby- distance is far.
**Governance:**	Where people face disadvantage due to ineffective, unjust, unaccountable or unresponsive global, national and/or sub-national institutions? Who is affected by inequitable, inadequate or unjust laws, policies, processes or budgets? Who is less or unable to gain influence or participate meaningfully in the decisions that impact them?	• What are the "Rules" , in place – are they fair and just?<br>• What are the informal rules within the household and who is benefiting from them?<br>• Are there forms of conflict resolution?<br>• Have they formal or informal rules that people abide by?<br>• How do socially ascribed identities determine decision making power?<br>• What factors weaken or reinforce social status?
**Socio-economic status:**	Those who face deprivation or disadvantages in terms of income, life expectancy and educational attainment. Those who have less chances to stay healthy, be nourished, educated and compete in the labour market? Those that can acquire wealth and/or benefit from quality health care, clean water, sanitation, energy, social protection and financial services?	• How are poverty and wealth perceived by in the local area?<br>• What aspects determine one's social status?<br>• How are women treated within the household, do they have their own money, have they access to land?
**Shocks and fragility:**	Those who are more exposed and/or vulnerable to setbacks due to the impacts of climate change, natural hazards, violence, conflict, displacement, health emergencies, economic downturns, price or other shocks?	• Are there hazardous elements known to be present in the are (e.g. water with high toxicity levels, nitrates)<br>• Is there a potential for extreme climate evens to happen in the area?<br>• Are the community vulnerable to these hazards (i.e. do they have no choice but to drink water, or they do not have adequate dwellings)<br>• Is there woman struggling to maintain their livelihoods due to discriminatory actions of others or the situation within their household?
1. Definitions taken from: **Leaving No One Behind** (LNOB) (UNDP, 2018)<br>2. Some characteristics in relation to gender inequalities taken from Manuel et al. (2024)		

S2. Sound boarding workshop

On the 14th of September 2021 an online- workshop was held where all REFOOTURE team members were present, approximately 20 people. Team members that participated were multidisciplinary and transdisciplinary action researchers. Several nationalities were present from South America, Africa and Europe. The purpose of the workshop was to reflect on the principles, to critique them and provide feedback on three major aspects: 1) reflect on principles, could/would people identify with the them? ; 2) what are the important aspects to consider for encouraging each principle to emerge from any process?; and 3) if the symbol resonated with them and potentially with the communities they were working? .

A Miro board was used to facilitate and capture the discussion, as well as the answers of the participants. The structure of the Miro reflected the 3 different locations where the project was active: Ethiopia, Kenya and Uganda.

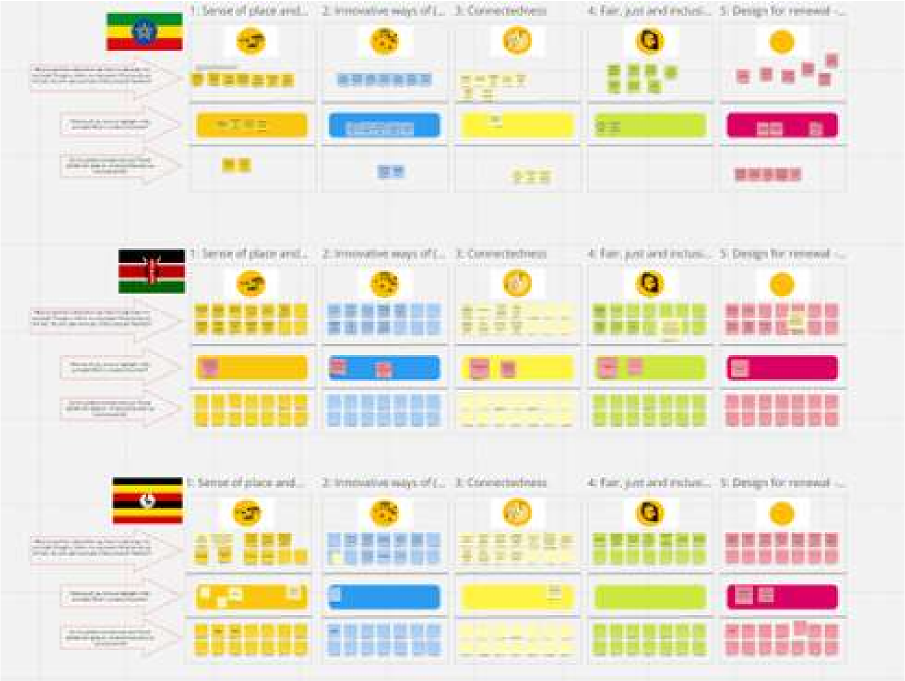

Figure S2.1 Screen shot of miro board used by each team working in the respective countries.

Results of the workshop

The results of the workshop are outlined in Table S2.1, summarising the key reflections and important considerations. While the symbols were identified as being useful to begin with, they are mostly to engage the scientific community in discussion (with a little support in understanding them). For the communities however, it was suggested that developing their own symbols for the RIFS principles could be used to simultaneously create ownership and awareness of the principles. This is particularly important if the literacy levels of communities engaged in the RIFS process are low. As a result of this workshop, Principle 4 was adapted to reflect the feedback received. Further additional literature and reading was also considered to check and capture the points raised.

Table S2.1 Summary of results from REFOOTURE Principles		
Principles	Key reflections/questions	Important considerations
**P1 Sense of Place and Purpose**	• Sense of purpose - what needs can fulfil the community – trigger them?<br>• There is something unique in every place – how to find it - nature people animal plans?<br>• Differences between spaces are important. We can learn from these differences.	• Do the activities conform to the local culture, practices and norms?<br>• How to build understanding of the players in that region (nature, people, culture) and how to contribute?<br>• How do we build capacities in place with the community and how will they recognise this potential?<br>• What needs to be regenerated?<br>• How to ensure this principles links to FNS?
**P2. Socio-Ecological Design for renewal**	• Giving room for indigenous technical knowledge that is with local communities<br>• Finding original ways of addressing food needs<br>• Finding balances between technological innovation and social innovation?<br>• Seeing (some) “problems” as “opportunities”<br>• Seeing potential of place	• Needs to be attractive to young generation who are more creative and innovative<br>• Innovative ways that are cost sensitive. Many farmers feel left behind because they don’t have financial means<br>• Tapping into relevant and specialised expertise to support the socio-ecological designs.
**P3. Building connections**	• Partnerships with other actors doing the same things - at the same level, do not work inn isolation<br>• What small actions can create big impact - ripple effects and strengthen connectedness?<br>• How to create trust, openness and trade-offs / compromises for the greater good?<br>• Do people see themselves being connected to a large system / biophysical system?<br>• How to build longer lasting relationships and (inter)actions?	• Producer influence on decision making - power relation in the food system<br>• A key point here - collaboration, sharing knowledge<br>• Stakeholders not always connected - though they are connected in the system<br>• Trade-offs in scales - consequences / repercussions of changes at larger or smaller scale
**P4. Fair, just and right relations**	• How to embrace differences between people/ stakeholder holders / diversity?<br>• How to ensure that nature/the environment is also part of decision making?<br>• How to ensure respect for local traditions and culture, if certain aspects need to be challenged?	• There is a need to support multi-level governance<br>• There is a need to ensure an equitable distribution of benefits<br>• Marginalised people with a big stake in the natural capital, but a poor in human and social capital need to be included - embrace the poorest<br>• How to empower women to create balance in the household, be gender sensitive in decision making?
**P5 Design for renewal it’s a living process**	• Is it working, is it not working - how to improve mechanism for giving feedback?<br>• Multiple stakeholders needed for reflection and continuous improvement<br>• Learning to learn from mistakes<br>• Create awareness and flexibility<br>• Institutionalising the process - permanent stakeholders to lobby for change	• Learning, co-creation, reflection<br>• Future generations need to be considered<br>• Thinking out of the box<br>• Do not starting from zero: principle should call on our responsibility for something; provide sources of inspiration; help to monitoring, reflection adaptability

S3. Summary table of literature reviewed to support the development of RIFS, frameworks, principles and values

Summary table of principles and values from regenerative development approaches and synergistic or complementary FS approaches and movements reviewed, organised according to the universal characteristics that were identified. In the cases where a value or principle is related to more than one characteristic it will appear under all associated characteristics.

Table S3.1 Guiding principles and values of regenerative approaches, synergistic and complementary food system approaches and movements

**Focus Area/Domain**	**Reference**	**Sense of place (Seeing the unique potential of place)**	**Collective purpose**	**Holistic and system thinking**	**Accountability and stewardship for innovations**	**Mutualistic connections and relationships (caring)**	**Autonomy and responsibility for decisions**	**Adaptive and collective learning**	**Designing for long term (renewal)**
FS/Academic[1]	Duncan et al., (2020)				Promoting accountable innovations<br><br>Moving beyond capitalist approaches	Taking care of people, animals, and the planet	Communing the food system	Acknowledging and including diverse forms of knowing and being	Long-term planning and rural-urban relations
Built Environs/ Regenerative practitioners Frameworks[2]	Plaut et al., (2012) CLEAR[3]	Focusing on Potential<br><br>Working from Uniqueness	Focusing on Potential<br><br>Spirit	Working with whole systems Interdependence Health	Nourishing Life Respecting limits	Being in reciprocal relationships partnership	Stewardship Justice		Intergenerational view
Developmental work/ Regenerative practitioners Frameworks	Mang and Reed, (2020)	Partner with place<br><br>Work from potential, not problems	Call forth a collective vocation<br><br>Find your distinctive value adding role	Leverage systemic regeneration by making nodal interventions	Design the design process to be developmental	Actualise stakeholder systems towards co-evolving mutualism			Design for evolution
ES/Academic[1]	Gibbons, (2020)[4]	Manifests potential in a place (potential-focused, not problem-focused) [b]		Works in whole systems (not fragments) [a]<br><br>Shifts thinking towards holistic worldview [a]	Value-adding: Contributes to healthier functioning/vitality of two next higher scales[c]	Mutualisms/Guilds: Creates reciprocal relationships that contribute to healthier, more vital whole[c]		Grows regenerative capacity (in human and more-than-human components of living systems—viability, vitality, evolutionary capacity)	Nodal leverage points: Identifies and shifts systemic leverage points to increase health and well-being[c]
Economics/Academic	Fath et al., (2019)		Promote mutually beneficial relationships and common-cause values	Maintain a healthy balance and integration of small, medium, and large organizations<br><br>Maintain robust, cross-scale circulation of critical flows including energy, information,	Promote constructive activity and limit overly extractive and speculative processes<br><br>Regenerative re-investment maintain a healthy balance of resilience and Efficiency	Promote mutually beneficial relationships and common-cause values		Promote effective, adaptive, collective learning	Maintain reliable inputs & healthy outputs<br><br>maintain a healthy balance of resilience and Efficiency

Table S3.1 Guiding principles and values of regenerative approaches, synergistic and complementary food system approaches and movements									
**Focus Area/Domain**	**Reference**	**Sense of place (Seeing the unique potential of place)**	**Collective purpose**	**Holistic and system thinking**	**Accountability and stewardship for innovations**	**Mutualistic connections and relationships (caring)**	**Autonomy and responsibility for decisions**	**Adaptive and collective learning**	**Designing for long term (renewal)**
				resources, and money<br><br>Maintain sufficient diversity					
Economics/ Regenerative practitioner[2]	Fullerton, (2015)	Honors Community and Place		Views Wealth Holistically	Innovative, Adaptive, Responsive<br><br>Robust Circulatory Flow	In Right Relationship<br><br>Edge effective abundance [5]	Empowered Participation	Edge effective abundance	Seeks Balance
SES/Academic [1]	Biggs et al., (2015)	Manage slow variables and feedback	Broaden participation *(to stimulate collective action)*	Foster complex adaptive systems thinking		Manage connectivity<br><br>Broaden participation (for building trust)	Broaden participation (the "right" people are involved)<br><br>Promote polycentric governance systems	Encourage learning and reflection	Maintain diversity and redundancy<br><br>Manage slow variables and feedback
Afforestation-FS/ Regenerative practitioner[2]	Maathai, (2010)	Gratitude and respect for Earth's resources	Love for the environment<br><br>Volunteerism		Accountability, transparency and honesty	Self and community empowerment	Accountability, transparency and honesty		
FS/Institutional (advocators of Agroecology)	FAO (2018)<br>HLPE (2019)	Culture and food traditions	Human and social values<br><br>Culture and food traditions	Synergies<br><br>Diversity	Co-Creation and sharing of knowledge<br><br>Circular and solidarity economy<br>Efficiency<br>Recycling	Human and social values<br><br>Circular and solidarity economy<br><br>Connectivity	Human and social values<br><br>Culture and food traditions<br><br>Responsible Governance	Co-Creation and sharing of knowledge	Resilience
Agriculture-FS/Regenerative agriculture practitioners	Rodale and Rodale M., (1989)	Potential (seeing the potential in self and around you)	Permanence (deeper meaning to life)	Pluralism (diversity)	Permanence (giving back to community)<br><br>Purity (without chemical inputs)	Peace (tolerance, compassion and understanding)	Protection (greater diversity in those active in the FS)	Pluralism (experiences and learning)<br><br>Progress	Protection (Resilience)

Table S3.1 Guiding principles and values of regenerative approaches, synergistic and complementary food system approaches and movements									
**Focus Area/Domain**	**Reference**	**Sense of place (Seeing the unique potential of place)**	**Collective purpose**	**Holistic and system thinking**	**Accountability and stewardship for innovations**	**Mutualistic connections and relationships (caring)**	**Autonomy and responsibility for decisions**	**Adaptive and collective learning**	**Designing for long term (renewal)**
								(capacity for well-being and enjoyment increases)	
Food Sovereignty/ Movement for agroecology	Nyéléni, (2015)	Territories are a fundamental pillar	Families, communities, collectives, organizations and movements are key	Agroecology is a way of life and the language of Nature	Women and their knowledge, values, vision and leadership are key<br><br>Production practices are based on ecological principles	Build alliances	Collective rights and access to the commons<br><br>Autonomy and self governance of communities<br><br>challenge and transform structures of power in society	The diverse knowledges and ways of knowing<br><br>Women and their knowledge, values, vision and leadership are key<br><br>radical space for young people to contribute to the social and ecological transformation	Equilibrium between nature, the cosmos and human beings.
Organic agriculture /advocators and practitioners of organic	IFOAM, (2020),	Ecology: Organic agriculture should be based on living ecological systems and cycles, work with them, emulate them and help sustain them.		Health: Organic agriculture should sustain and enhance the health of soil, plant, animal, human and planet as one and indivisible.	Care: Organic agriculture should be managed in a precautionary and responsible manner to protect the health and well-being of current and future generations and the environment.	Fair: Organic agriculture should build on relationships that ensure fairness with regard to the common environment and life opportunities.			Ecology: Organic agriculture should be based on living ecological systems and cycles, work with them, emulate them and help sustain them.

1. Academic here refers to participatory action research - meaning the insights are based on participatory process and experiences from researchers mostly at academic institutes. Using second order research, which according to Kok et al., (2019) is where the "rresearcher is engaged in the system of interest and tries to understand and transform it". They are part of the transformation process themselves.
2. Regenerative practitioner refers to institutes such as the Regenesis Institute (https://www.regenerat.es/) or the CLEAR institute (Centre for Living Environments and Regeneration: https://www.clearegeneration.org/) or the Capital institute (https://capitalinstitute.org/regenerative-capitalism/) , the Green Belt movement (https://www.greenbeltmovement.org/) which are active in area of mobilizing communities for regenerative developmental work. In some cases the institutes are also using and establishing different regenerative tools to support such work, e.g. LENSES approach developed by CLEAR (https://www.clearegeneration.org/lenses/) (Plaut et al., 2012; Plaut et al., 2016), The regenerative practitioner (TRP) series where the frameworks of Mang and Reed, (2020) and Mang and Haggard (2016) are taught.
3. These principles are a combination of both sources: CLEAR can be found here, https://www.clearegeneration.org/free-sources/
4. Meta principles are defined by letters with a) referring to wholeness, b) referring to change and c) referring to relationships
5. Edge effective abundance refers to "stepping *outside one's usual silos leads to tremendous bursts of creativity, synergy, and satisfaction…. a regenerative economy works hard to find ways to cultivate common cause synergy and cross-fertilization at the "edges*."

S4. Theoretical examples of how principles can be used across the three lines of work

This section provides illustrative examples of how the authors envision the three lines of work to be used in conjunction with the principes, based on authors' own observations and experiences. While written from the perspective of the people described e.g. researcher, community member, employee, these insights are based on other work and discussions which the authors have had with pioneers and "doers" in the food system. Naturally, these are simple descriptions and do not digress into the potential nuances or challenges associated with the examples. They may, in some cases be classified as idealised in nature, however they are presented here with the purpose of stimulating reflection on these topics and to encourage further discussion and deliberation.

**Principle 1 Sense of Place and Purpose (P1)**			
**Description**	**Examples first line: Developing oneself**	**Examples second line: Growing the capability and motivation of the community or team**	**Examples third line: Improving the health and value of a system**
Regenerative Inclusive Food Systems start with people, and with our unique experience of the places where we live. Through caring for each other and for the unique character of the places where we live, we can nourish ourselves while experiencing ourselves as part of a community of living species and systems that together make up a place.	*Researcher[1]:* Be open to experiential learning through actively listen to the community and their needs, be genuine about wanting to create transformative change	Build capacities in the community that empower them to see for themselves their own potential and that of their place, the role that they can play in enhancing the functional traits of the place (e.g. food, livelihoods) while maintaining the spirit and nature of the place	Identify the stakeholders that create enabling/disabling conditions for people and place and encourage them to also see the potential of the place and the role they could play in enhancing and changing aspects of the local FS that will allow for regenerative outcomes (e.g. the Four RIFS outcomes)[2]
	Community member: Be open to rekindle or build your connection to your home community and place to see all the potential that is already there and can be grown	Reflect on how you want to support the community, what do you want to bring to the community, that everyone has the potential to create positive change in the practices done in the community, how things are organized and how people interact with nature and the place	Find ways to co-learn and share your learning and skills with your neighbors and those from outside the community and place. Enjoy the increased vitality of place and your role as one of the important stewards of the community and for helping regenerative outcomes to emerge (e.g. the Four RIFS outcomes)
	International company (mid- level employee): Be open to experiential learning through actively listen to the community (who lives were raw material is sourced) to understand how business interests and relationships can be built on trust could be more than extraction, it could play a more powerful role	Within the capacity of your position identify what it is you can do to support the regenerative aspirations of the community.	Find others in the hierarchy of the company with similar convictions to create positive changes within the company in how materials are sourced, using the experiential learning from community and placed based visits
1. Researcher involved in a placed-based RIFS project<br>2. Linked to the four RIFS outcomes areas: Ecosystem Health, Social Equity and Caring communities and Food and Nutritional Security			

**Principle 2 Socio-ecological design for innovation (P2)**			
Description	**Examples first line: Developing oneself**	**Examples second line: Growing the capability of the community or team**	**Examples third line: Improving the health and value of a system**
These are triggered by the needs of a community, where innovators are inspired to find solutions and opportunities in using the available resources and local knowledge in more effective and creative ways, while working with nature for the collective well-being of all.	*Researcher[1]:* Find innovative ways of engaging with pioneers, change makers and communities (P3) that are doing socio-ecological innovative actions in their production/consumption approaches. Challenge yourself to be non-extractive in collecting evidence (P4), to make the connections/integration of socio-ecological effects (positive or negative) use this to support the innovators to inform the transformation to RIFS	Build the capacities also within the community to collect and observe the evidence that empowers them and encourages them to learn, adapt and change their activities to reap the positive effects of their socio-ecological innovations and efforts (P5)	Create the scientific underpinning that can allow other researchers to contribute to building solid scientific evidence to show how socio-ecological innovations can support the regeneration of communities and places. Show both the benefits and also potential trade-offs that this can bring. Support the co-development of frameworks and tools that can stimulate and capture the potential of socio-ecological innovations and the learnings from associated change processes that can enhance RIFS further
	Community member: Instead of using the paradigms of problem-solutions finding within your community, try to look for creating opportunities out of challenges. Start from a positive orientation. Try to find ways that can couple livelihoods of the community, fairness, as well as nature and food	Find others also with similar ideas and form a group to collectively encourage and support each other, create opportunities for peer-to-peer learning, co-learn and develop socio-ecological innovations that can support the community and the regeneration of the FS in place (linked to P3, P5)	Be conscious to observe and record the changes occurring with socio-ecological innovations being used to create and support the RIFS outcomes, use lessons learned to inform decisions (P5), be curious about further opportunities to enhance the community and the four outcome areas of RIFS
	International company (mid- level employee): Observe the manner in which products are produced at the company, could they be more innovative? Respecting and giving back to the communities and culture of the area where the materials are sourced. Are there possibilities of using aspects of nature to enhance the production to need less resources and create positive impact? Try to find ways to bring this thinking approach into the company	Is there a possibility to talk with the company product designers (P3)? To help them understand how a potential change in raw material properties need to have adapted processes or designs (due to more regenerative practices e.g. fiber length change, lower sugar content). Is there a way to also do so while taking into account the use of resources, as a first step using opportunities of circularity to support improved design process. Starting small with a pilot project (Small wins)	With results from pilot projects and the experiential learning (P5), can these be used to show, management or advocators within the leadership of the company of the potential business opportunities and corporate social and environmental responsibility actions that reconfiguring supply chains could create. They could become leading stewards championing change and transformation towards RIFS
1. Researcher involved in a placed-based RIFS project			

Principle 3: Building connections (P3)			
	**Examples first line: Developing oneself**	**Examples second line: Growing the capability of the community or team**	**Examples third line: Improving the health and value of a system**
Building connections with each other and with nature makes us stronger, together we become better able to cope with, adapt to, and even consciously initiate system changes.	*Researcher[1]:* Work on personal connections and relationships to the nature around you. Connect to other staff members at your institute that have a desire for creating meaningful change, regardless of background and positionality. Challenge each other and learn each others language to create a common understanding, as this is invaluable. (1st order research)	Connect with real life change makers and their networks, find ways to include them in projects, to encourage transdisciplinary action, co-development and support them and their actions to connect with nature/communities helping also to underpin this work with scientific processes (2nd order research)	If the situation allows act as a facilitator or a type of broker between communities and power holders, creating an even playing field, where all are understood to have a role in the future of a place and all have a responsibility to ensure the health of the community and place.
	Community member: Work on personal connection and relationship to the nature around you. Connect with active members in the community, that have a similar purpose (P1) in creating regenerative results, within your neighborhood or locality. Try still to connect and understand those with a different point of view to understand where aspects can be reconciled.	If possible, link people in your community or encourage others to find connections between members, so that together greater support can be created for one another e.g. peer -to -peer learning for innovative ideas on producing food or other products and services or supports that are useful for the community. Connect to those innovators outside of the locality which may also bring valuable insights and learning that are useful for creating regenerative results.	Once a collective community purpose has been found and ambitions and visions for regenerative results in your location (P1) can be communicated, reach out to those whose decisions can make real long-lasting change, connect with the power holders and build a working relationship with them. As a community connect to other communities and share the actions and learnings you have to, encourage others to develop similar thinking, approaches and begin to learn from others too.
	International company (mid- level employee): Work on person connections and relationships to the nature around you. Connect with like minded people in your organisation. Be open to hearing the potential for creating meaningful and useful connections that can help to build a strong network of employees that seek regenerative results at the company	Connect people within your network that can help to create positive momentum within the organisation for regenerative practices. Recognize when there is a need for concrete data and connect those with that data to those that need it – to show the positive benefits of regenerative business practices.	Connect with other like-minded people outside the organisation and in similar areas of operation. If possible, share results experiential learning, to show others interested how regenerative actions can lead to results that can create positive business opportunities.

1. Researcher involved in a placed-based RIFS project

**Principle 4. Fair, just and inclusive transition (P4)**			
Description	**Examples first line: Developing oneself**	**Examples second line: Growing the capability of the community or team**	**Examples third line: Improving the health and value of a system**
In a regenerative, inclusive food system, justice, fairness and inclusivity are the foundations for promoting responsibility, accountability and giving a voice to all those involved in the production and consumption of food, including nature.	*Researcher[1]:* Try to be aware of potential unconscious bias. Be conscious to include the relevant ideas and people, encourage yourself to actively listen. Have empathy and respect in your dealing with others.	Use the knowledge and skill of the team and the community to empower themselves to create change on the ground. Don't be afraid to question the sources of "common terms" and approaches that do not appear to be fair or inclusive, or even encourage research practices or scientific dialogue in a manner that does not contribute to creating a fair and inclusive society[2]	Help the communities in the places where the regenerative project is based to, find the "Win-Win-Win" strategies (Win for them, win for nature and win for power holders e.g. those that can make system changing decisions; government, companies, institutes).
	Community member: Try to be aware of potential unconscious bias, why there is an orientation towards one group over another. If events are being organized, try to include the relevant people. Aim to actively listen and be open to new ideas and approaches	Empower the community to see their true value and their potential for also steering and deciding on their own futures and the future of their home place and their food system. Aim to include those especially seen as disadvantaged and gave them the opportunity to be equals.	Use the momentum for change to also create a level playing field for all parties involved. This will allow more socially equitable conditions. Make sure nature has a seat at the table in all of the discussions and collaborations with power holders and community (Win-win-win)
	International company (mid- level employee): Try to be aware of potential unconscious bias or predetermined expectations of actors or suppliers in the companies supply chain. Try to be conscious of how business is being conducted, does it give back, contribute to communities	If there are clear areas for potential improvements in fairness of contracts, negotiation approaches or engagement with local partners, strive to support the creation of more fair and transparent approaches that create better conditions for the places where the company is sourcing its raw material	Create win-win-win momentum for the company, community and nature. Added value is not always monetary. Find the threshold where other "values" or capitals (e.g. natural, human, cultural) compensate for lack of financial gain, experiential learning (P1-3) of advocators within the company can also help to determine these thresholds.

1. Researcher involved in a placed-based RIFS project
2. Reiterating some questions found in the main manuscript "Are both scientific and traditional agroecological knowledge treated the same and, if not, why not? Furthermore, are there disparities between the benefits of knowledge and research money going to the global north, while many of the issues are occurring in the global south? How do we equalize our roles for tackling these knowledge gaps together?

**Principle 5 Design for renewal (it's a living process) (P5)**			
Description	**Examples first line: Developing oneself**	**Examples second line: Growing the capability of the community or team**	**Examples third line: Improving the health and value of a system**
Transitioning towards regenerative and inclusive food systems is a living process that emphasizes learning by doing, building the capabilities needed to continue to regenerate these systems as conditions evolve, so that ongoing sustenance can be provided to human and natural communities.	*Researcher[1]:*<br>Try to take time to reflect on the process, to reflect on what has been learnt. Reflect on the situations how is success measured, how to handle impatience.<br>Try to use lessons learned to improve the next cycle of work striving for regenerative results, RIFS, in the next project undertaken	Try to allow for learning, especially learning from mistakes. Learn how to work and be in dynamic interactions between a diverse team. Learn how to communicate between specialists in the team as well as generalists.<br>Try to write proposals that allow some time for reflection and sensemaking of complex process and transformation processes	Become advocators of "think-do-reflect-adapt" in projects to support regenerative results. Using the scientific learning and acumen built up, as a network of researchers working within both scientific institutions and real life, challenge the academic norms for conducting research to answer, "societies grand challenges" (3rd order research)[2]
	Community member:<br>Try to take time to reflect on the process, to reflect on has been learnt. Reflect on the situations how is success measured, how to handle impatience. Try to use lessons learned to improve the next cycle of work striving for regenerative results within the community and home place	The work of the community is never finished as RIFS requires continuous engagement. The composition of the community group may change over time. Regular reflection and revision of what the group has achieved, does and still wants to collectively achieve, helps to keep the sense of purpose and curiosity for learning by doing alive	Become an advocator of "think-do-reflect-adapt" in community projects using the other 4 RIFS principles to underpin such actions P1-P4. Reflect on the principles have they supported RIFS results for the community and the home place? What lessons can be drawn to support others to build momentum for change
	International company (mid- level employee):<br>Try to take time to reflect on the process, to reflect on has been learnt. Reflect on the situations how is success measured, how to handle impatience. Try to use lessons learned to improve the next cycle of work striving for regenerative results within your company	If possible, within the organisation, explore if there is some space for experiential learning- learning by doing. Use small pilot projects to create examples and help you and others to understand how regenerative can work at a small scale, to understand how it can be scaled (up and deep)	With results from pilot projects and the experiential learning (P5), can these be used within the company to create greater awareness of the company's potential for regenerative change? Within the company with like minded people, encourage a culture of reflection on the potential role the company can take with regards to regenerative results and win-win-wins (P4)

1. Researcher involved in a placed-based RIFS project

2.Using Kok et al., (2019) description of third order research "*perspective: the researcher is engaged in the system of interest as well as in the research and innovation system..acknowledges this multisystemic complexity and aims to understand and transform both systems by changing their interaction*" or by being active in creating within the research and innovation system an enabling environment that supports more transdisciplinary work

S5. Examples of how principles are being used to guide research and participatory approaches being implemented within REFOOTURE

Support questions to help teams in the field with active listening, making observations and reflecting after community engagement sessions.

Questions Sense of place and purpose

- What makes the place unique? What are the specific resources and circumstances of that place?
- What do people care about in that place? What is the relationship that people have with nature?
- What are people proud of? What positively surprises other people when they come to this place?
- What makes people curious? What triggers innovation?
- What's the energy of the place, community like?

Questions socio-ecological design for innovation

- What practices are they doing to produce, prepare and consume food, are these regenerative (many positive benefits) or degenerative for the community for the place?
- What happens with waste in the area, especially food and animal wastes, are there issues?
- What sources of fuel are they using, is this collected harvested, produced in a degenerative manner?
- If there is a drought/flood – is the food system able to buffer these effects? Is there a tendency towards vulnerability, scarcity?
- What types of technologies are being used? Are other technologies not being used, being considered/desired, would these types fit the place/community?
- What nature-based solutions/practices stand out? Where did this knowledge come from?
- How are they financing these initiatives? Are they feasible?
- How do practices/services of the innovation benefit social equity to what extent?
- Can you see any other potential opportunities that are currently not being explored?
- Are there cross cutting issues that can turn issues into opportunities?

Questions building connections

- What are the relationships like in the groups/community? Are they jovial, distant?
- Social cohesion within the group, is there a culture of trust and common purpose between these different people or is it more a business type relationship?
- What are people saying about nature, the place, can you establish their connection to place, to particular locations? Are these derived from childhood, familial connections, religious connections, resources use?
- Do they have system thinking, can they connect their activities to something bigger?
- Are there advocators for change (a driving force) involved in bringing people together?

Questions Fair, Just and Inclusive

- How are the benefits or burdens of food production, preparation and consumption being shared? Is it fair, within the household, within the community? What roles are women playing, what roles are men playing, children?
- How does the community homesteads look, good infrastructure – are there elements of disadvantages visible?
- Based on your entry points into the community (e.g. local representatives, advocators or pioneers of change within a community) can you identify

- Which people are commonly excluded for what reasons? Are there entry points into these communities
- Are people able to think about the future or are they living from day -to-day? Have they space to dream?
- Are people afraid to speak out, or they speak quite freely without any fear?
- Are there dominant people with negative energy?

Questions Learning for Renewal (Living process)

- Are people learning by doing, testing out ideas – can they afford to make mistakes, or would it be too costly for them?
- How are success stories (and failures to learn from) conserved and shared?
- Do people have the curiosity and the drive to keep learning and improving?
- Have people a sense of how their place works i.e., they almost know when the weather will change and what they need to do to buffer the effects? (generational learning)

References

Biggs, R., M. Schlüter, M.L. Schoon. 2015. Eds. Principles for building resilience: sustaining ecosystem services in social-ecological systems. Cambridge University Press, Cambridge.

Burkhard, B., F. Müller, A. Lil. 2008. Ecosystem Health Indicators. In *Encyclopaedia of Ecology*, eds. S.E. Jørgensen, B.D. Fath. 1132-1138. Oxford: Academic Press.

Costanza, R., and M. Mageau 1999. What is a healthy ecosystem? *Aquatic Ecology* 33: 105-115. https://doi.org/10.1023/A:1009930313242.

De Steenhuijsen Piters, B., E. Termeer, D. Bakker, H. Fonteijn, andn H. Brouwer. 2021. Food system resilience. Towards a joint understanding and implications for policy. No. 2021-077. Wageningen Economic Research.

Duncan, J., M. Carolan, and J.S.C Wiskerke. 2020. Routledge Handbook of Sustainable and Regenerative Food Systems, Rural Sociology Group, Wageningen University, Netherlands.

FAO. 2018. The 10 elements of agroecology. Guiding the transition to sustainable food and agricultural systems.

FAO. 2019. TAPE Tool for Agroecology Performance Evaluation 2019 – Process of development and guidelines for application. Test version. Rome. Available at: https://openknowledge.fao.org/items/8511c796-c7d1-4a04-895d-a28115731ce0.

FAO- ITPS. 2020. Intergovernmental Technical Panel on Soils. Towards a definition of soil health. Soil Letters no. 1. Available at: https://www.fao.org/global-soil-partnership/itps/itps-soils-letter/en/.

Fath, B.D., D.A. Fiscus, S.J. Goerner, A. Berea, and R.E. Ulanowicz. 2019. Measuring regenerative economics: 10 principles and measures undergirding systemic economic health. *Global Transitions* 1: 15-27. https://doi.org/10.1016/j.glt.2019.02.002.

Fullerton, J. 2015. Regenerative Capitalisim. How Universal Principles And Patterns Will Shape Our New Economy. White paper. Available at: https://capitalinstitute.org/regenerative-capitalism/.

Gibbons, L.V. 2020. Regenerative—The New Sustainable? *Sustainability* 12: 5483. https://doi.org/10.3390/su12135483

Gibbons, L.V., G. Pearthree, S.A. Cloutier, and M.M Ehlenz. 2020. The development, application, and refinement of a Regenerative Development Evaluation Tool and indicators. *Ecological Indicators* 108: 105698-105698. https://doi.org/10.1016/j.ecolind.2019.105698

HLPE. 2019. Agroecological and other innovative approaches for sustainable agriculture and food systems that enhance food security and nutrition. A report by the High-Level Panel of Experts on Food Security and Nutrition of the Committee on World Food Security, Rome.

HLPE. 2020. Food security and nutrition: building a global narrative towards 2030. A report by the High-Level Panel of Experts on Food Security and Nutrition of the Committee on World Food Security, Rome.

Ifejika Speranza, C., U. Wiesmann, and S. Rist. 2014. An indicator framework for assessing livelihood resilience in the context of social–ecological dynamics. *Global Environmental Change* 28: 109-119. https://doi.org/10.1016/j.gloenvcha.2014.06.005

IFOAM. 2020. Principles of organic agriculture. Available at: https://www.ifoam.bio/sites/default/files/2020-03/poa_english_web.pdf.

Jacobi, J., S. Mukhovi, A. Llanque, H. Augstburger, F. Käser, C. Pozo, M. Ngutu Peter, J.M.F. Delgado, et al. 2018a. Operationalizing food system resilience: An indicator-based assessment in agroindustrial, smallholder farming, and agroecological contexts in Bolivia and Kenya. *Land Use Policy* 79: 433-446. https://doi.org/10.1016/j.landusepol.2018.08.044

Kok, K.P.W., A.C.L. Den Boer, T. Cesuroglu, M.G. van der Meij, R. de Wildt-Liesveld, B.J. Regeer, and J.E.W. Broerse. 2019. Transforming Research and Innovation for Sustainable Food Systems—A Coupled-Systems Perspective. Sustainability 11: 7176. https://doi.org/10.3390/su11247176

Maathai W.2010. Replenishing the earth: Spiritual values for healing ourselves and the world. Crown Publishing Group, New York.

Mang, P., B. Haggard, Regensis. 2016. Regenerative Development: A Framework for Evolving Sustainability. John Wiley & Sons, Ltd.

Mang, P., and B. Reed. 2020. Regenerative Development and Design. In: *Loftness*, Sustainable Built Environments. eds. V. Hasse, D. 115-141. New York: Springer US. https://doi.org/10.1007/978-1-4614-5828-9_303

Manuel, J., M.G. Rivera-Ferre, F. López-i-Gelats. 2024. Contributions of a feminist perspective to the analysis of farm viability: the livelihoods reproduction framework. *The Journal of Peasant Studies* 51: 185-211,10.1080/03066150.2023.2210500.

Narasimhan, T.N., 2009. Hydrological Cycle and Water Budgets. In *Encyclopedia of Inland Waters,* eds. G.E. Likens. Elsevier, Oxford, pp.. 714-720.

Nyéléni (2015) Declaration of the International Forum for Agroecology, Nyéléni, Mali: 27 February 2015. Development 58, 163-168.

Plaut, J., B. Dunbar, H. Gotthelf, and D. Hes. 2016. Regenerative Development through LENSES with a case study of Seacombe West. *Environment Design Guide*, 1-19.

Plaut, J.M., B. Dunbar, A. Wackerman, and S. Hodgin. 2012. Regenerative design: the LENSES Framework for buildings and communities. *Building Research & Information* 40: 112-122. https://doi.org/10.1080/09613218.2012.619685

Reemer, T., S. O'Keeffe, B. De Groote, P. Murage, D. Kigiri, and P. Ndambiri. 2023. "Collaborate to Regenerate" A guide for teams to start mobilising people towards regenerative and inclusive food systems. Available on line at: https://www.wur.nl/en/project/refooture-food-futures-eastern-africa.htm.

Robertson, D.M., H.A. Perlman, T.N., and Narisimhan, T.N., 2022. Hydrological Cycle and Water Budgets. In *Encyclopedia of Inland Waters (Second Edition),* eds. T. Mehner, K. Tockner, K.19-27. Oxford: Elsevier.

Rodale, B., and Rodale M. (1989) Seven tendencies towards regeneration in agriculture, communities and personal spirit. Available at: https://rodaleinstitute.org/wp-content/uploads/7-TENDENCIES-REGENERATION.pdf.

Schulte, R.P.O., R.E. Creamer, T. Donnellan, N. Farrelly, R. Fealy, C. O'Donoghue, and D. O'hUallachain. 2014. Functional land management: A framework for managing soil-based ecosystem services for the sustainable intensification of agriculture. *Environmental Science & Policy* 38: 45-58,https://doi.org/10.1016/j.envsci.2013.10.002.

Shu-Yang, F., B. Freedman, and R. Cote. 2004. Principles and practice of ecological design. *Environmental Reviews* 12: 97-112. https://doi.org/10.1139/a04-005

Sundkvist, Å., R. Milestad, and A. Jansson. 2005. On the importance of tightening feedback loops for sustainable development of food systems. *Food Policy* 30: 224-239.

UNDP. 2018. United Nations Development Programme. What does it mean to leave no one behind? A UNDP discussion paper and framework for implementation. Available at: https://www.undp.org/publications/what-does-it-mean-leave-no-one-behind. Accessed: April 2021.

Vollmer, D., K. Shaad, N.J. Souter, T. Farrell, D. Dudgeon, C.A. Sullivan, I. Fauconnier, G.M. MacDonald, et al. 2018. Integrating the social, hydrological and ecological dimensions of freshwater health: The Freshwater Health Index. *Science of The Total Environment* 627: 304-313. https://doi.org/10.1016/j.scitotenv.2018.01.040
